# Supplementary material for: Translational regulation by Hfq–Crc assemblies emerges from polymorphic ribonucleoprotein folding
Source: EMBO J. 2022 Dec 12;42(3):e111129. doi: 10.15252/embj.2022111129 (PMC9890229; doi:10.15252/embj.2022111129)
Supplement: Supplementary file 1 — Expanded View Figures PDF [file EMBJ-42-e111129-s001.pdf]

Expanded View Figures

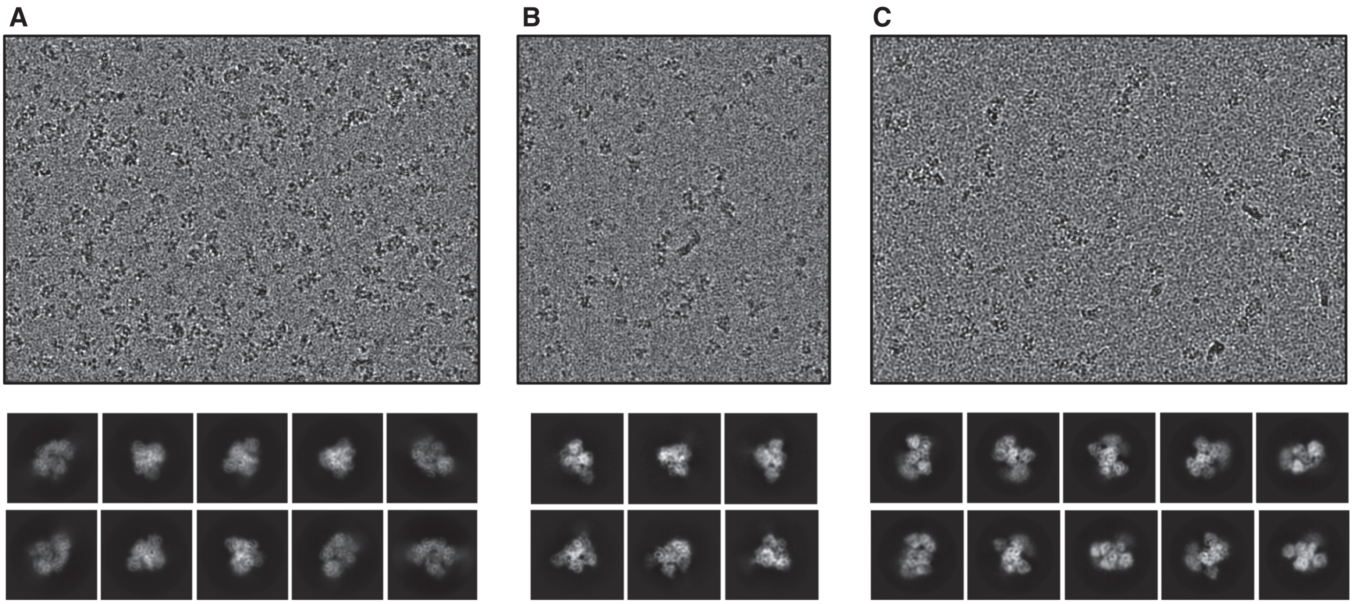

Figure EV1. Raw images and 2D class averages of the Hfq–Crc translation repression complexes formed on *amiE*$_{105}$ (A), *estA*$_{118}$ (B), and *rbsB*$_{110}$ (C).

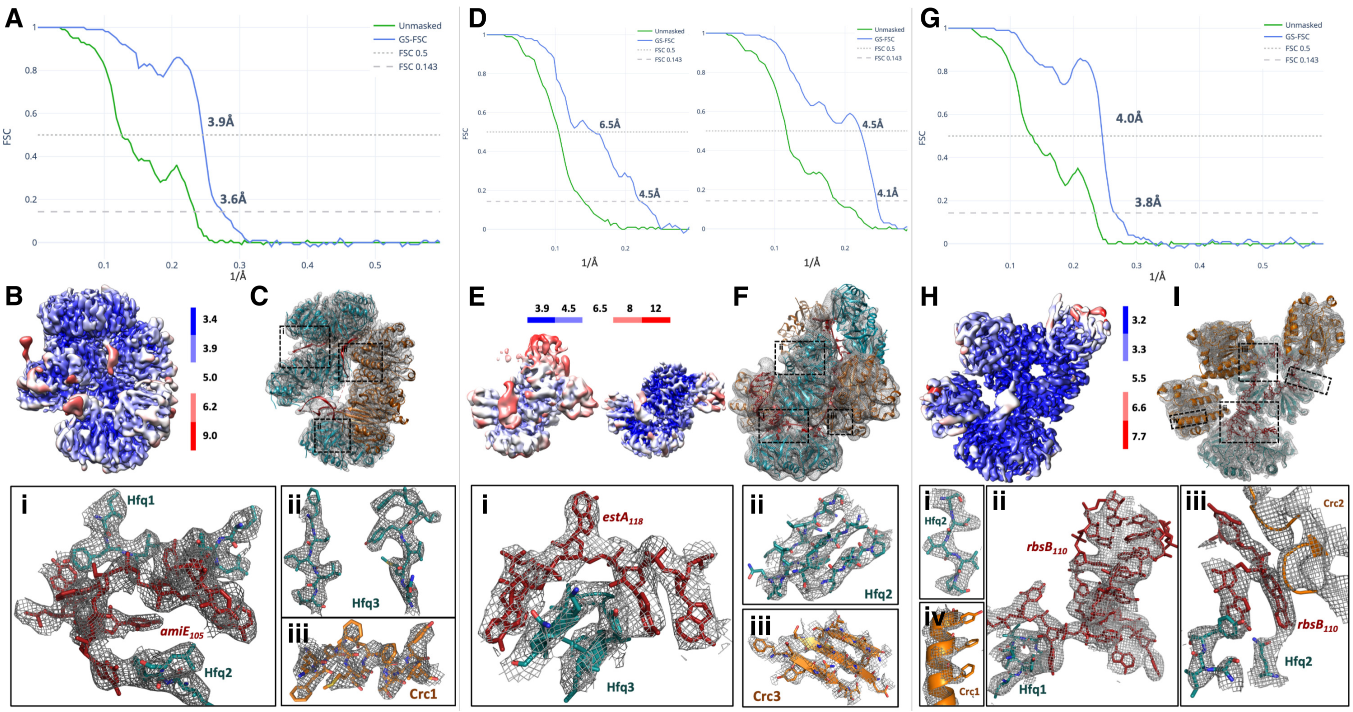

Figure EV2.

© 2022 The Authors

Figure EV2. Global and local resolution analyses of the Hfq–Crc translation repression complexes formed on *amiE*$_{105}$, *estA*$_{118}$, and *rbsB*$_{110}$.

A–I (A), (D), and (G) show global FSC curves (gold standard) for the *amiE*$_{105}$, *estA*$_{118}$, and *rbsB*$_{110}$ complexes, respectively. The left FSC curve in panel (D) corresponds to the global, consensus refinement; the right FSC curve corresponds to the focused refinement for the Hfq–estA118–Crc reconstruction. (B), (E), and (H) display local resolution estimates as measured by cryoSPARC at FSC 0.5. (C), (F), and (I) show the respective structures, colored as before, docked into the experimental cryo-EM maps, with the insets showing close-up of selected areas for each.

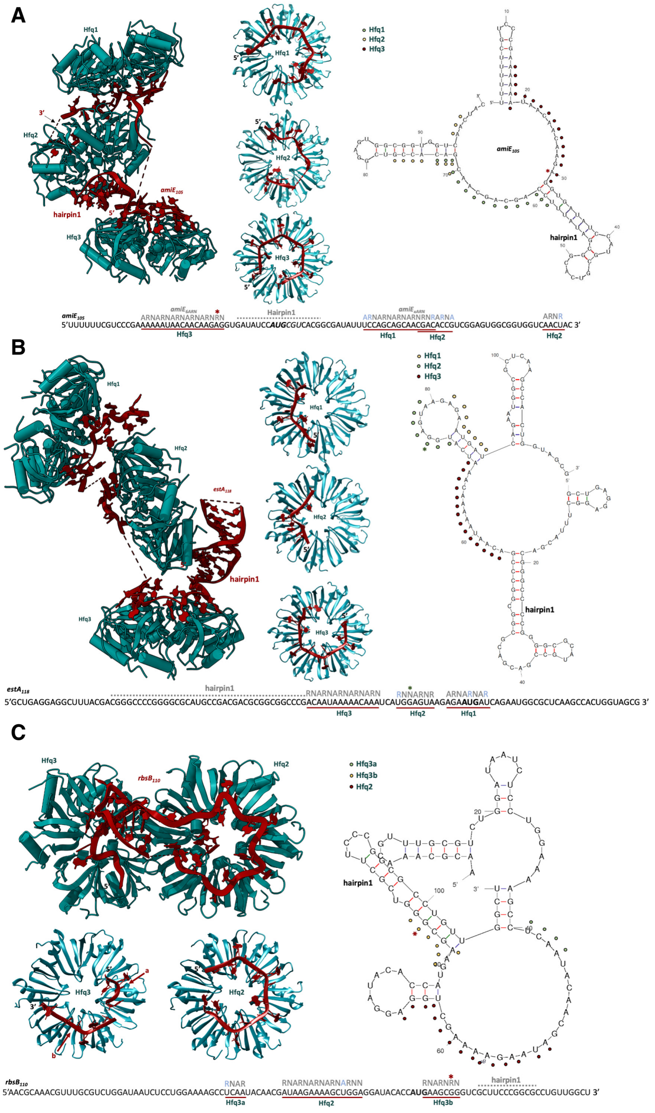

Figure EV3. Recognition and presentation of *amiE*$_{105}$, *estA*$_{119}$, and *rbsB*$_{110}$ by Hfq.

A *amiE*$_{105}$ is presented by three Hfq hexamers and adopts complete or partial ARN motif engagement on each distal side. The proximal side of Hfq2 coordinates an amiE105 hairpin-loop structure (hairpin1). A second hairpin-loop forms at the 3′-end of amiE105 on the Hfq2 distal side (hairpin2, not shown due to limited resolution). Right: annotated secondary structure prediction of *amiE*$_{105}$ (mfold; Zuker, 2003). Colored dots indicate which Hfq distal side presents the ARN-rich motif in the Hfq–*amiE*$_{105}$–Crc model. An annotated sequence is depicted at the bottom of the panel. Sequences that were mapped in the cryo-EM reconstruction are underlined in red and the Hfq distal sides they bind to are labeled in green. Occupied A-, R-, or N-sites are annotated in gray above each modeled sequence. * refers to an A-site "skipping"-violation, where the A-site on the Hfq distal site is not occupied by a base, i.e., skipped. Light blue letters refer to "mismatch"-violations of the ARN rule, where a pyrimidine base occupies an A-site or R-site pocket on the Hfq distal face. The ranges for hairpin1 and hairpin2 are arbitrary due to limited local resolution in the corresponding map regions.

B *estA*$_{118}$ is presented by three Hfq hexamers and adopts partial ARN motif engagement on each Hfq distal side. The proximal side of Hfq2 coordinates an estA118 hairpin-loop structure (hairpin 1). Right: annotated secondary structure prediction of *estA*$_{118}$ (mfold; Zuker, 2003). Colored dots indicate which Hfq distal side presents the ARN-rich motif in the Hfq–Crc–*estA*$_{118}$ model. An annotated sequence is depicted at the bottom of the panel. Sequences that were mapped in the cryo-EM reconstruction are underlined in red and the Hfq distal sides they bind to are labeled in green. Occupied A-, R-, or N-sites are annotated in gray above each modeled sequence. The * refers to an A-site "skipping"-violation, where the A-site on the Hfq distal site is not occupied by a base, i.e., skipped. Light blue letters refer to "mismatch"-violations of the ARN rule, where a pyrimidine base occupies an A-site or R-site pocket on the Hfq distal face.

C *rbsB*$_{110}$ is presented by three Hfq hexamers (only the two that were well resolved in the cryo-EM maps are shown) and adopts partial ARN motif engagement on each Hfq distal side. The proximal side of Hfq2 coordinates a *rbsB*$_{110}$ hairpin-loop structure (hairpin1, in the back of the Hfq2 hexamer, not annotated in the figure). Right: annotated secondary structure prediction of *rbsB*$_{110}$ (mfold; Zuker, 2003). Colored dots indicate which Hfq distal side presents the ARN-rich motif in the Hfq–*rbsB*$_{110}$–Crc model. An annotated sequence is depicted at the bottom of the panel. Sequences that were mapped in the cryo-EM reconstruction are underlined in red and the Hfq distal sides they bind to are labeled in green. Occupied A-, R-, or N-sites are annotated in gray above each modeled sequence. * refers to an A-site "skipping"-violation, where the A-site on the Hfq distal site is not occupied by a base, i.e., skipped. Light blue letters refer to "mismatch"-violations of the ARN rule, where a pyrimidine base occupies an A-site or R-site pocket on the Hfq distal face. The range for hairpin1 is arbitrary due to limited local resolution in the corresponding map region.
