# Supplementary material for: Translational regulation by Hfq–Crc assemblies emerges from polymorphic ribonucleoprotein folding
Source: EMBO J. 2022 Dec 12;42(3):e111129. doi: 10.15252/embj.2022111129 (PMC9890229; doi:10.15252/embj.2022111129)
Supplement: Supplementary file 2 — Table EV1 [file EMBJ-42-e111129-s002.docx]

Table EV1: **Cryo-EM data collection and refinement statistics for the Hfq/Crc/RNA structures.**

| Structure | Hfq- *amiE_105_-Crc* | Hfq- *estA_118_-Crc* | Hfq-*rbsB_110_-Crc* |
| --- | --- | --- | --- |
| PDB code | XX | XX | XX |
| EMDB code | XX | XX | XX |
| Data collection |  |  |  |
| Microscope | FEI Titan Krios G2 | FEI Titan Krios G2 | FEI Titan Krios G2 |
| Voltage (kV) | 300 | 300 | 300 |
| Detector | Gatan K3 | Gatan K3 | Gatan K3 |
| Nominal magnification | 105 000x | 105 000x | 105 000x |
| Pixel size (Å) | 0.83 | 0.83 | 0.83 |
| Electron dose, per frame (e^–^/Å^2^) | 1.03 | 1.02 | 1.03 |
| Defocus range (µm) | -1.1 / -2.5 | -1.1/2.5 | -1.1 / -2.5 |
| Exposure (s) | 1.9 | 1.9 | 1.9 |
| Number of micrographs | 14996 | 5532 | 6674 |
| Reconstruction |  |  |  |
| Software | cryoSPARC 2.15 | cryoSPARC 2.15 | cryoSPARC 2.15 |
| Number of particles used | 70 572 | 61945 | 148739 |
| Final resolution, FSC_0.143_ (Å) | 3.6 | 4.4 (4.1) | 3.8 |
| Map-sharpening B factor (Å^2^) | -100 | -128 | -117 |
| Model |  |  |  |
| Composition (Hfq:Crc:RNA) | 3:4:1 | 3:4:1 | 3:3:1 |
| Non-hydrogen atoms | 20109 | 19452 | 17218 |
| Protein residues | 2269 | 2266 | 1979 |
| RNA nucleotides | 83 | 53 | 52 |
| Molar Mass (kDa) | 317 | 322 | 288 |
| Refinement |  |  |  |
| Software | Refmac5/Phenix/  Isolde | Refmac5/Phenix/  Isolde | Refmac5/Phenix/  Isolde |
| Correlation coefficient, masked | 0.81 | 0.77 | 0.75 |
| FSC_0.5_ (model-map) | 3.9 | 7 | 3.6 |
| Validation |  |  |  |
| MolProbity score | 1.67 | 2.1 | 1.50 |
| Clash score | 5.06 | 8.5 | 2.80 |
| Ramachandran |  |  |  |
| Favoured, overall (%) | 94.02 | 93.56 | 95.71 |
| Allowed, overall (%) | 5.75 | 6.08 | 3.67 |
| Outlier, overall (%) | 0.22 | 0.36 | 0.63 |
| R.m.s. deviations |  |  |  |
| Bond length (Å) | 0.015 | 0.015 | 0.015 |
| Bond angle (°) | 1.8 | 1.7 | 1.9 |
