# Supplementary material for: Translational regulation by Hfq–Crc assemblies emerges from polymorphic ribonucleoprotein folding
Source: EMBO J. 2022 Dec 12;42(3):e111129. doi: 10.15252/embj.2022111129 (PMC9890229; doi:10.15252/embj.2022111129)
Supplement: Supplementary file 3 — PDF+ [file EMBJ-42-e111129-s003.pdf]

Article

TRANSPARENT PROCESS
OPEN ACCESS

THE EMBO JOURNAL

Translational regulation by Hfq–Crc assemblies emerges from polymorphic ribonucleoprotein folding

Tom Dendooven[1], Elisabeth Sonnleitner[2], Udo Bläsi[2] & Ben F Luisi[1,*]

Abstract

The widely occurring bacterial RNA chaperone Hfq is a key factor in the post-transcriptional control of hundreds of genes in *Pseudomonas aeruginosa*. How this broadly acting protein can contribute to the regulatory requirements of many different genes remains puzzling. Here, we describe cryo-EM structures of higher order assemblies formed by Hfq and its partner protein Crc on control regions of different *P. aeruginosa* target mRNAs. Our results show that these assemblies have mRNA-specific quaternary architectures resulting from the combination of multivalent protein–protein interfaces and recognition of patterns in the RNA sequence. The structural polymorphism of these ribonucleoprotein assemblies enables selective translational repression of many different target mRNAs. This system elucidates how highly complex regulatory pathways can evolve with a minimal economy of proteinogenic components in combination with RNA sequence and fold.

Keywords co-transcriptional RNA folding; Crc; metabolic regulation; ribonucleoprotein assembly; RNA chaperone Hfq; translational regulation
Subject Categories Microbiology, Virology & Host Pathogen Interaction; Structural Biology; Translation & Protein Quality
DOI 10.15252/embj.2022111129 | Received 10 March 2022 | Revised 25 October 2022 | Accepted 11 November 2022 | Published online 12 December 2022
The EMBO Journal (2023) 42: e111129

Introduction

RNA-binding proteins play central roles in post-transcriptional control of gene expression. In the pathogenic bacterium *Pseudomonas aeruginosa*, one major regulatory system depends on the CsrA-like Rsm proteins, which act as translational repressors of target mRNAs (Dubey *et al*, 2005; Schubert *et al*, 2007; Goodman *et al*, 2009; Holmqvist *et al*, 2016; Romero *et al*, 2018; Gebhardt *et al*, 2020). Another main system depends on the RNA chaperone Hfq, a member of the widely occurring Lsm/Sm protein family, which plays numerous roles including facilitating the actions of small regulatory RNAs (sRNAs; Pusic *et al*, 2021), acting as a translational repressor of target mRNAs (Sonnleitner & Bläsi, 2014; Kambara *et al*, 2018; Sonnleitner *et al*, 2018; Malecka *et al*, 2021), and supporting ribosome biogenesis (Andrade *et al*, 2018). Through these activities, Hfq contributes to the coordination of stress responses (Lu *et al*, 2016), metabolism (Sonnleitner & Bläsi, 2014), quorum sensing (Sonnleitner *et al*, 2006; Yang *et al*, 2015), virulence (Sonnleitner *et al*, 2003), and affects complex processes such as biofilm formation and antibiotic susceptibility (Fernandez *et al*, 2016; Pusic *et al*, 2016, 2018; Zhang *et al*, 2017; Sonnleitner *et al*, 2020; Trouillon *et al*, 2022), and linking translational repression to stress-induced mutagenesis (Chen & Gottesman, 2017). Hfq can interact with other proteins to achieve these *in vivo* functions (dos Santos *et al*, 2019; Dendooven *et al*, 2021).

Hfq-mediated translational repression forms the basis for a hierarchical control of carbon and nitrogen utilization by *Pseudomonas* spp., a mechanism referred to as carbon catabolite repression (CCR; Rojo, 2010; Sonnleitner & Bläsi, 2014). CCR ensures that preferred carbon sources, such as succinate, are used before alternative nutrients are utilized. The regulation is exerted through translational repression of genes affecting the uptake and metabolism of non-preferred nutrients (Sonnleitner & Bläsi, 2014). One well-studied CCR-regulated gene is *amiE*, which encodes the enzyme aliphatic amidase that generates organic acids from short-chain aliphatic amides, thereby enabling *Pseudomonas* to utilize acetamide as a source of both carbon and nitrogen. When preferred carbon sources such as succinate are abundant, translation of *amiE* mRNA is suppressed through sequestration of the ribosome-binding site by Hfq and the catabolite control protein Crc (Fig 1A), which is followed by mRNA degradation (Sonnleitner & Bläsi, 2014). When the preferred carbon source is exhausted, CCR is alleviated by the regulatory sRNA CrcZ (Fig 1A), which sequesters Hfq away from substrate mRNAs (Sonnleitner & Bläsi, 2014). CrcZ levels are controlled by the alternative sigma factor RpoN (Sonnleitner *et al*, 2009; Abdou *et al*, 2011; Valentini *et al*, 2014) and the two-component system CbrA/B, which may be activated in response to the cellular energy status (Valentini *et al*, 2014).

Understanding the molecular basis of CCR has been advanced by structural and functional insights into RNA recognition by Hfq

1 Department of Biochemistry, University of Cambridge, Cambridge, UK
2 Department of Microbiology, Immunobiology and Genetics, Max Perutz Labs, University of Vienna, Vienna, Austria
*Corresponding author. Tel: +44 7914 093485; E-mail: bfl20@cam.ac.uk

© 2022 The Authors. Published under the terms of the CC BY 4.0 license.

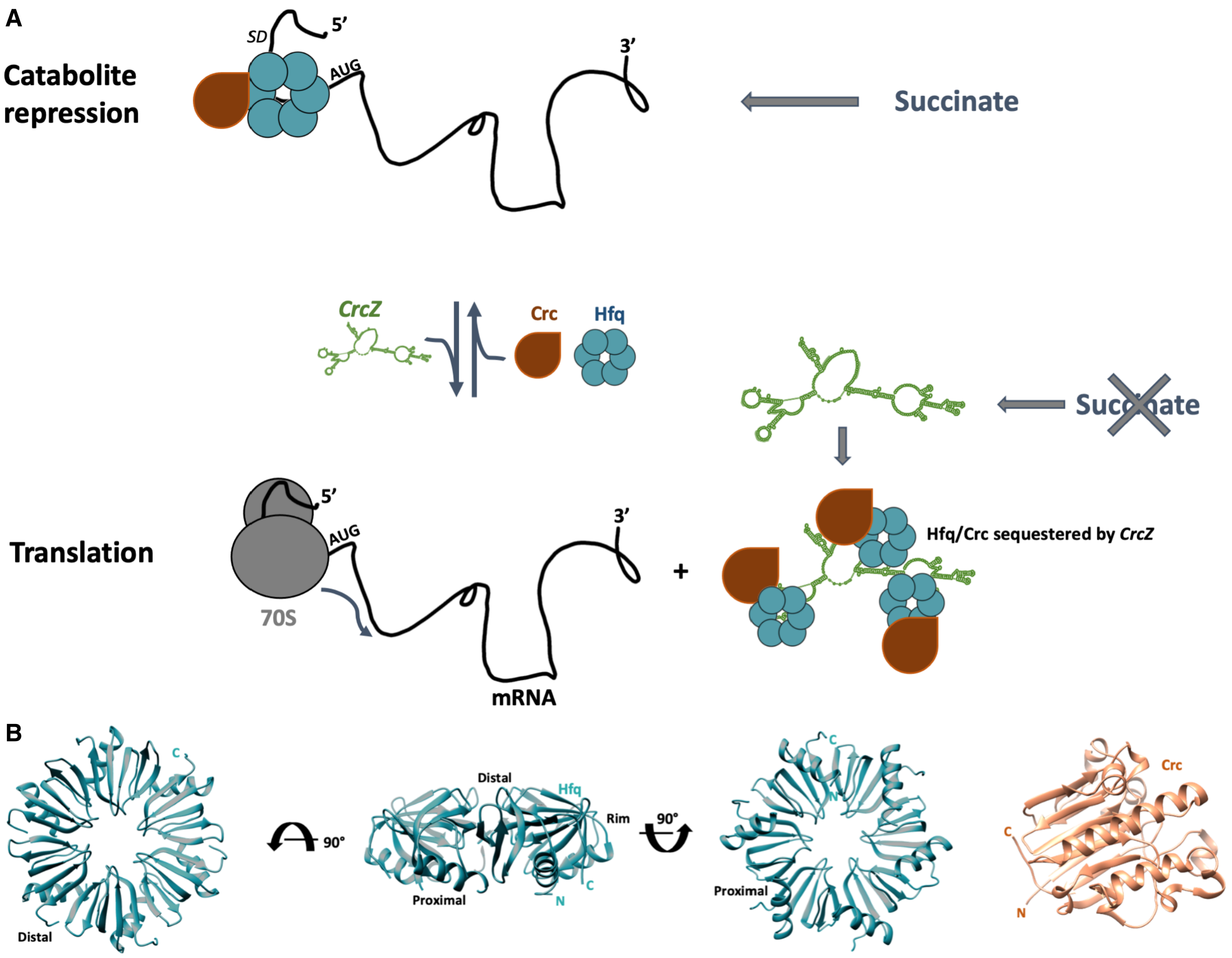

Figure 1. Catabolite repression in *Pseudomonas* spp.

A During catabolite repression, e.g., when succinate levels are high (top), the Hfq hexamer and Crc cooperatively bind to A-rich sequences at the 5′-end of target mRNAs and mask the ribosome-binding site. When succinate levels are low (bottom), catabolite repression is alleviated by the regulatory RNA CrcZ, which sequesters Hfq and/or Hfq/Crc complexes.

B Crystal structures of the Hfq hexamer, showing the distal, proximal, and rim side (left three panels), and Crc (right panel).

(Zhang *et al*, 2013; Santiago-Frangos & Woodson, 2018). These have identified three different RNA-binding surfaces on the Hfq hexamer: the proximal face, the distal face, and the circumferential rim (Fig 1B). The proximal face binds uridine tracts, which are enriched at the 3′ end of sRNAs, the distal face has sequence preference for ARN triplet motifs (where A is an adenosine, R is a purine, and N is any base), and the rim has arginine-rich patches that can interact with UA-rich motifs of RNAs (Santiago-Frangos & Woodson, 2018). How the Pseudomonad-specific Crc protein contributes to the repressive function of these CCR assemblies has been a long-standing question. Cryo-EM structures of Hfq–Crc complexes on a short octadecameric segment derived from the 5′ upstream untranslated region (5′-UTR) of *amiE* mRNA (Fig 2A; $amiE_{6ARN}$) revealed how the Hfq distal side presents the *amiE* ribosome-binding site to Crc (Pei *et al*, 2019). In these structures, four Crc protomers are sandwiched between two Hfq hexamers, each of which present one $amiE_{6ARN}$ motif to Crc. The structures suggested that translation repression complexes are higher order assemblies, where several Hfq hexamers and Crc molecules are engaged on the mRNA target. Interestingly, Crc has no intrinsic RNA-binding capacity and does not interact with Hfq in the absence of a RNA substrate (Milojevic *et al*, 2013; Sonnleitner *et al*, 2018). Thus, the ability of Crc to engage Hfq–mRNA intermediates arises through the cooperative effects of the interactions in the higher order assemblies. In line with this model, single-molecule fluorescence assays and molecular dynamics simulations showed that Crc interacts with transient, pre-organized Hfq/RNA complexes and shifts the equilibrium toward assemblies with increased stability (Krepl *et al*, 2021; Malecka *et al*, 2021). Thus, the cooperation of Hfq with Crc stabilizes the repressive complex, excluding the 30S ribosomal subunit more effectively than Hfq alone.

 © 2022 The Authors

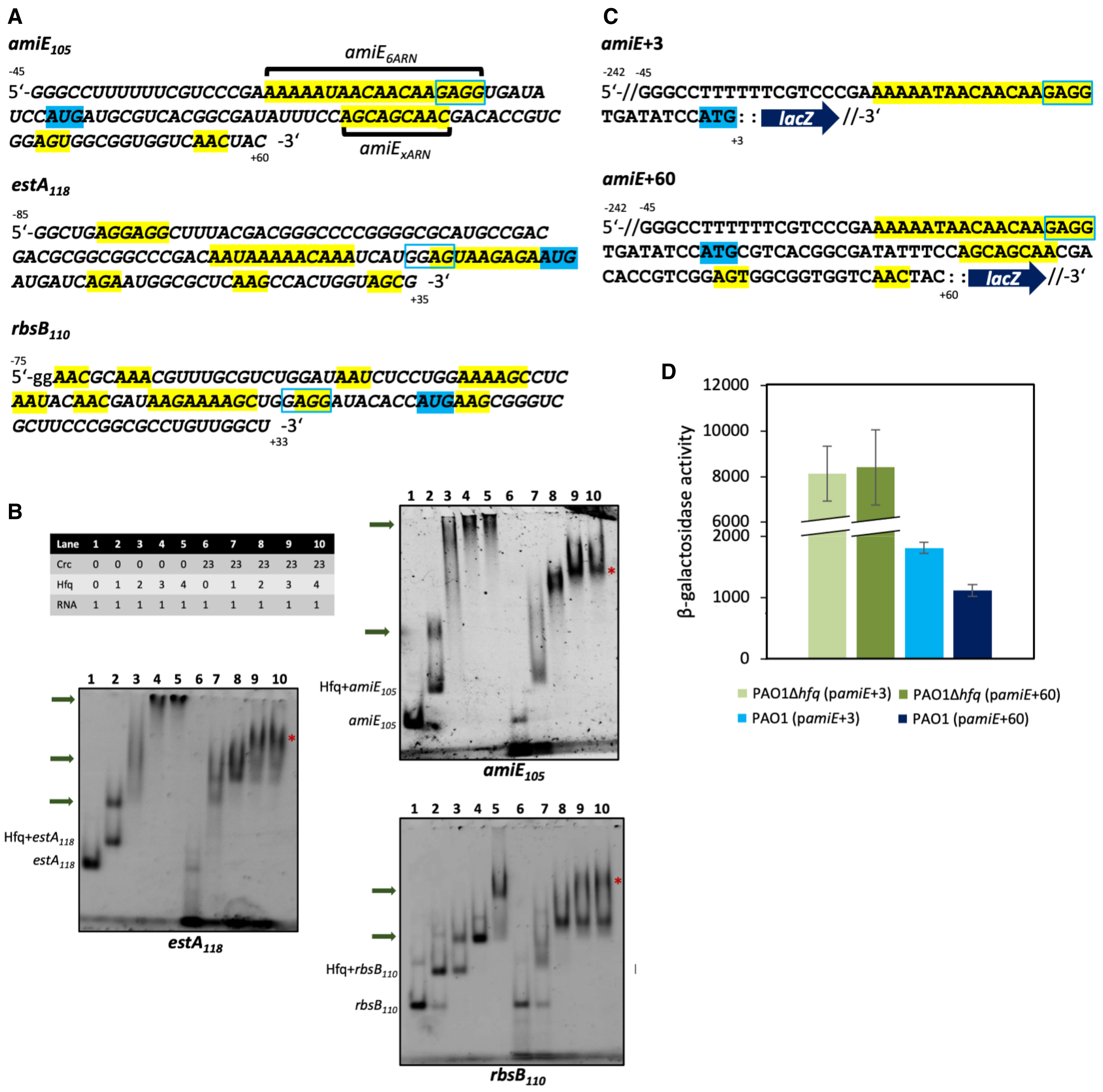

Figure 2. Higher order Hfq/Crc assemblies form on *amiE*$_{105}$, *rbsB*$_{110}$, and *estA*$_{118}$ mRNA segments.

A Sequences of the 5′UTRs and the proximal coding regions of *amiE*$_{105}$, *rbsB*$_{110}$, and *estA*$_{118}$ mRNA fragments. ARN motifs are highlighted in yellow, Shine–Dalgarno sequences are highlighted in blue rectangles, and the AUG start codons are highlighted in blue. The *amiE*$_{6ARN}$ segment used by Pei *et al* (2019) is annotated as well as the shorter, secondary ARN$_{xARN}$ segment in the *amiE* coding region.

B Electrophoretic mobility shift assays (EMSA) with *amiE*$_{105}$, *estA*$_{118}$, and *rbsB*$_{110}$ transcripts in the presence of Hfq alone and Hfq/Crc. Multiple Hfq molecules can engage a single RNA molecule (lanes 1–5), and Hfq and Crc can form compact higher order assemblies on all RNA targets tested (lanes 6–10). Top: The table shows relative stoichiometries in the samples (RNA is at 200 nM). The protein components were mixed first, after which the RNA fragments were added. Green arrows highlight Hfq–RNA oligomers, and the red asterisks (*) highlight the highest order complexes for the stoichiometry range tested.

C Two translational *amiE*::*lacZ* reporter genes were constructed to test the downstream ARN cluster of *amiE in vivo*. The *amiE*+3::*lacZ* reporter gene contained a 29-nucleotide long *amiE* fragment encompassing the 5′UTR with the six ARN motifs (*amiE*$_{6ARN}$) and the AUG translation initiation codon fused to *lacZ*. The longer *amiE*+60::*lacZ* construct included additional 57 nucleotides downstream of the ATG start codon that encompass the second ARN-rich motif.

D When compared with the expression in a *P. aeruginosa* strain lacking Hfq (PAO1Δ*hfq*), the translation of the *amiE*+60::*lacZ* gene in the PAO1 wt strain was repressed significantly stronger than that of the *amiE*+3::*lacZ* gene. These *in vivo* results support the structural data presented in Fig 4A and B in that the ARN-rich motif in the coding region of *amiE* can contribute to the formation of effective Hfq–Crc repressive complexes in addition to the proximal *amiE*$_{6ARN}$ motif.

© 2022 The Authors

Recent RNA-seq and proteomics approaches have revealed that CCR controls more than 100 mRNA targets that are co-regulated by Hfq and Crc, many of which are involved in carbon metabolism and virulence (Corona *et al*, 2018; Kambara *et al*, 2018). However, the diverse regulatory functions of Hfq/Crc present a puzzling issue: how can the same two effector molecules target so many different mRNA sequences with specificity and individually tuned response? To explore this question, we solved the cryo-EM structures of Hfq/Crc translation repression complexes assembled on extended 5′-end segments of the *amiE*, *estA*, and *rbsB* genes, encoding an amidase, an esterase, and a putative ribose transporter, respectively (Winsor *et al*, 2016), and we present *in vivo* observations supporting our models. These structures revealed how multiple ARN repeats in the RNA targets are engaged by Hfq and Crc to form higher order repressive complexes. The results expand the repertoire of higher order complex ribonucleoprotein assemblies that involve Hfq (dos Santos *et al*, 2019; Dendooven *et al*, 2021). Strikingly, the Hfq/Crc/RNA complexes are polymorphic in quaternary organization. The determinants for the organization are a combination of RNA secondary structure elements and the position and length of the ARN motifs in the *t*ranslation *i*nitiation *r*egion (TIR) of these transcripts. Permissive Crc dimerization and small, versatile Hfq–RNA–Hfq interfaces further stabilize the diverse repressive assemblies. The polymorphic character of these complexes enables Hfq and Crc to target many genes, while maintaining sequence specificity. These findings define a new paradigm for *in vivo* action of Hfq through cooperation with the Crc helper protein to form diverse RNA-driven effector assemblies that regulate expression of numerous target genes.

Results

Higher order Hfq/Crc assemblies form on mRNA targets *in vitro*

Based on our earlier cryo-EM structures of a complex formed by Hfq and Crc on an 18-mer element from the 5′-UTR of the *amiE* transcript (Pei *et al*, 2019), we hypothesized that higher order assemblies may form on longer mRNA segments that contain multiple ARN motifs in the TIR. Here, we studied Hfq/Crc assembly on mRNA fragments derived from the TIRs of the *amiE*, *estA*, and *rbsB* genes, all of which were previously shown to be regulated by Hfq and Crc (Sonnleitner & Bläsi, 2014; Kambara *et al*, 2018). As shown in Fig 2A, the $amiE_{6ARN}$ sequence present in the 5′UTR of the *amiE* gene is followed by one cluster of three complete ARN motifs in the immediate coding region, the $amiE_{xARN}$ motif, and two ARN triplet motifs further downstream. These downstream ARN motifs have been implicated in Hfq binding *in vivo* by proximity crosslinking of Hfq and DNA in nascent transcripts followed by Hfq-specific chromatin immunoprecipitation coupled with DNA sequencing (ChIP-seq; Kambara *et al*, 2018). In contrast to *amiE* RNA, clusters of ARN motifs are predominantly found in the 5′-UTR of *rbsB* and *estA* mRNA, rather than in the immediate 5′coding region (Fig 2A). These regions are anticipated to engage Hfq which is in accord with the reported ChIP-seq data (Kambara *et al*, 2018). To verify that higher order assemblies form on different mRNA targets *in vitro*, electrophoretic mobility shift assays were performed with the mRNA fragments $amiE_{105}$ (nts −45 to +60), $rbsB_{110}$ (nts −75 to +33), and $estA_{118}$ (nts −85 to +33; Fig 2B). For all three transcripts, a number of higher order species was observed to form with increasing Hfq concentrations (Fig 2B, lanes 1–5). In the presence of excess Crc, formation of higher order species plateaued at around three Hfq hexamers per RNA target for all mRNA fragments tested (Fig 2B; lanes 7–10). These observations support the hypothesis that multiple Hfq hexamers can engage different ARN-rich motifs in an mRNA target and form defined species in the presence of Crc.

Sequential formation of higher order Hfq–Crc assemblies from a core complex

To characterize the details of the oligomeric state and quaternary structure, the Hfq–Crc complex formed on $amiE_{105}$ was analyzed by cryo-EM. For the grid preparation, a Hfq-titration series was performed like that used for the EMSAs shown in Fig 2B. Fig 3A shows a gallery of the key species observed in the titration series. Although it is possible that the pathway for the formation of the higher order complexes may be heterogenous, based on the observed species we propose a pathway where in the first step a Hfq–Crc–Crc core is formed, whereby the $amiE_{6ARN}$ region is bound by the Hfq distal side with two Crc molecules recognizing and engaging the Hfq–RNA complex (Fig 3A, left, 1:1:2 Hfq:$amiE_{105}$:Crc). From this core, another Hfq and Crc can bind to form a higher order intermediate (Fig 3A, middle, 2:1:3 Hfq:$amiE_{105}$:Crc), and in a final step, a third Hfq engages the intermediate assembly, together with a fourth Crc molecule (Fig 3A, right, 3:1:4 Hfq:$amiE_{105}$:Crc). The structure of the 3:1:4 Hfq:$amiE_{105}$:Crc complex was solved at 3.6 Å resolution (Figs 3B and EV1A and EV2A–C). This complex is proposed to fully mask the *amiE* 5′-end to prevent translational initiation. We noted that the Crc/Crc dimerization interface seen in the previous 2:2:2 complex with the short *amiE* RNA octadecamer ($amiE_{6ARN}$; Pei *et al*, 2019) is not present in the Hfq–Crc complex formed on $amiE_{105}$. The Crc:Crc interface seen earlier appears to be disrupted due to the longer *amiE* RNA, which permits an Hfq hexamer (Hfq2) to fit into the space (Fig 3A and B) that was filled with the dimerizing Crc/Crc pair in the Hfq:$amiE_{6ARN}$:Crc assembly (Pei *et al*, 2019).

Hfq–Crc complexes are polymorphic in quaternary structure

To further elucidate the architectural principles of Hfq/Crc repressive complexes, we solved structures of Hfq–Crc complexes formed on the TIRs of the $estA_{118}$ and $rbsB_{110}$ mRNA segments (Fig 2A). Representative images and 2D class averages are shown in Fig EV1B and C. The reconstructions were generated at 4.4 Å and 3.8 Å resolution, respectively (Figs 3C and D, and EV2D–I). Focused refinements resulted in a 4.1 Å resolution reconstruction of a subassembly of the Hfq:$estA_{118}$:Crc complex (Figs 3C; right inset and EV2E). In both complexes, the mRNA target sequences are bound by three Hfq hexamers and three ($rbsB_{110}$) or four ($estA_{118}$) Crc molecules (Fig 3C and D). Notably, we observed different quaternary structures for each mRNA target, $amiE_{105}$, $estA_{118,}$ and $rbsB_{110,}$ demonstrating the assembly of polymorphic Hfq–Crc RNPs, driven by the mRNA sequence (Fig 3B–D and EV3). The core interactions of Crc/Hfq seen in the 1:1:2 complex in Fig 3A remain the same as in the earlier reported 2:2:2, 2:4:2, and 2:3:2 complexes with the *amiE* octadecamer ($amiE_{6ARN}$; Pei *et al*, 2019), which appears to be

 © 2022 The Authors

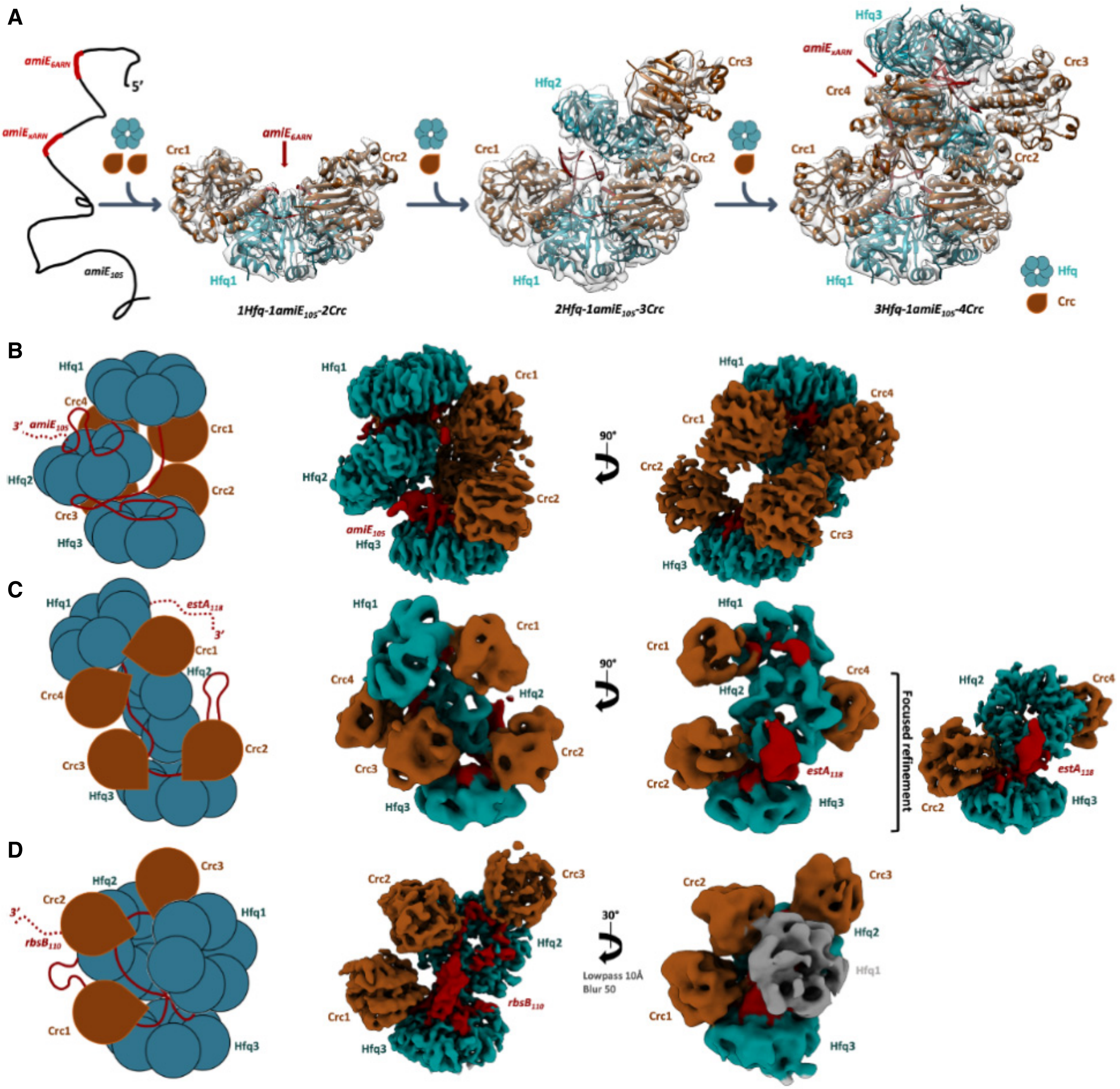

Figure 3. Hfq–Crc translation repression assembly pathway, and complexes formed on *amiE*$_{105}$, *estA*$_{118}$ and *rbsB*$_{110}$.

A A proposed cooperative assembly pathway for Hfq–Crc assemblies on *amiE*$_{105}$. The *amiE*$_{6ARN}$ region (Fig 1A) is bound by the Hfq distal side. Subsequently, two Crc molecules recognize and engage the Hfq–RNA complex (left). An additional Hfq and Crc can then bind to form a higher order intermediate (middle). In a final step, a third Hfq engages the intermediate assembly, together with a fourth Crc molecule (right). The final complex fully masks the *amiE*$_{105}$ 5′-end and is proposed to inhibit translation initiation.

B Schematic and cryo-EM map of the *amiE*$_{105}$ translation repression complex where two Hfq hexamers (Hfq1 and 3) enclose four Crc molecules (Crc1–4) and a third Hfq hexamer (Hfq2). The *amiE*$_{105}$ fragment threads through the complex, engaged by all three Hfq hexamers and all four Crc molecules.

C In the *estA*$_{118}$ complex, two Hfq hexamers (Hfq1 and 3) enclose four Crc molecules (Crc1–4) and a third Hfq hexamer (Hfq2), like the *amiE*$_{105}$ complex. The *estA*$_{118}$ fragment threads through the assembly and contacts all three Hfq hexamers and all four Crc molecules. Hfq1 and Crc1 are flexibly tethered to the translation repression complex and were excluded during local refinements (right panel; see Fig EV2). Global Hfq–Crc–*estA*$_{118}$ maps were low-pass filtered to 9 Å to aid visualization (middle two maps). The locally refined map is shown at 4.1 Å resolution (right).

D Three Hfq hexamers (Hfq1–3) present the *rbsB*$_{110}$ mRNA to three Crc molecules (Crc1–3). The density for Hfq1 is diffuse, which may be explained by flexible association of Hfq to the translation repression complex.

© 2022 The Authors

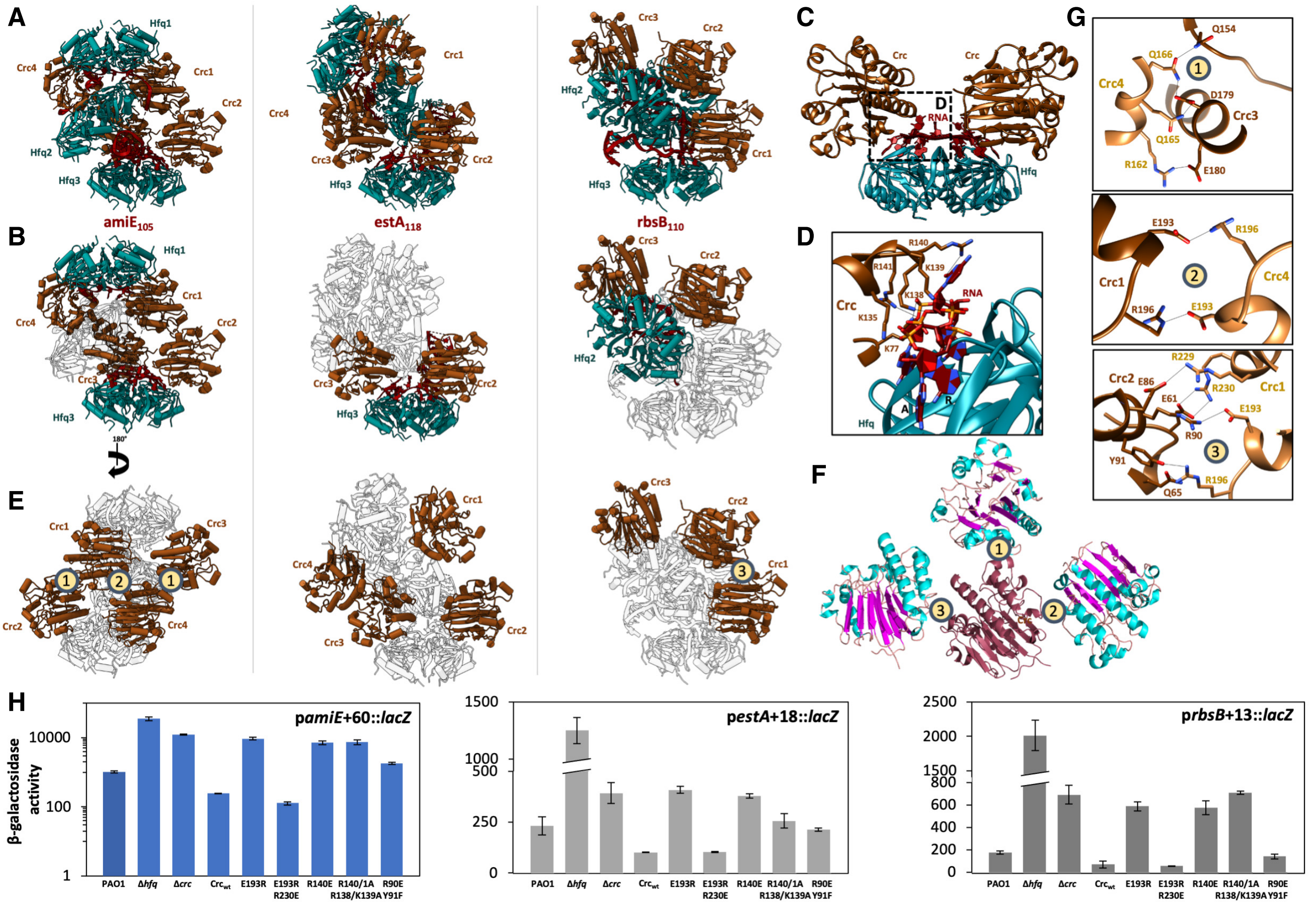

Figure 4. Conserved RNA binding by Crc and diverse protein-to-protein interfaces between Crc protomers in the Hfq–Crc assemblies on *amiE*$_{105}$, *estA*$_{118}$, and *rbsB*$_{110}$.

A–C All three assemblies are built from one or two Hfq–Crc–Crc core complexes, where Hfq presents ARN-rich motifs to two Crc molecules. The full repressive assemblies formed on *amiE*$_{105}$, *estA*$_{118}$, and *rbsB*$_{110}$ are depicted in panel A. In panel B, the same complexes are depicted, but only the Hfq–Crc–Crc core units are colored. In panel C, the Hfq-Crc-Crc core structure is depicted.

D A close-up of the Crc–RNA interactions in the core units. All Crc molecules in the repressive assemblies interact with the RNA via the same binding mode. A basic patch on the Crc surface, comprised of K77/K135/K138/K139/R140/R141, engages the phosphate backbone and a few N-site bases of the presented RNA on the Hfq distal side.

E–G Crc forms homodimers in the *amiE*$_{105}$ and rbsB$_{110}$ assemblies via diverse permissive dimer interfaces. Panel E shows the full repressive assemblies formed on *amiE*$_{105}$, *estA*$_{118}$, and *rbsB*$_{110}$ where only the Crc molecules are colored. Three Crc dimer interfaces were observed in the *amiE*$_{105}$ and *rbsB*$_{110}$ complexes, annotated as 1, 2, and 3. Panel F shows the three Crc dimers observed, aligned, and superimposed on a reference Crc molecule, with the interfaces annotated as in panel E. A close up of each dimer interface is given in panel G.

H Translational regulation of *amiE*+60::*lacZ*, *rbsB*+13::*lacZ*, and *estA*+18::*lacZ* reporter genes by Crc variants. *P. aeruginosa* strain PAO1Δcrc, harboring either plasmids p*amiE*+60::*lacZ* (H; left panel), p*estA*+18::*lacZ* (H, middle panel), p*rbs*+13::*lacZ* (H, right panel), or plasmid pME4510 (vector control) together with pME4510*crc*$_{Flag}$ (Crc$_{wt}$) or derivatives thereof encoding the respective mutant proteins was grown to an OD$_{600}$ of 2.0 in BSM medium supplemented with 40 mM succinate and 40 mM acetamide (p*amiE*+60::*lacZ*) or 40 mM succinate alone (p*estA*+18::*lacZ* and p*rbsB*+13::*lacZ*). The β-galactosidase activity values conferred by the corresponding LacZ fusion proteins are indicated. The results represent data from two independent experiments and are shown as mean and range.

the fundamental unit upon which higher order assemblies form. From these structures, recurring features can be observed that define architectural principles of Hfq/Crc assembly during CCR.

In all three Hfq/Crc complexes with *amiE*$_{105}$, *estA*$_{118}$, and *rbsB*$_{110}$, the ARN-rich motifs in the mRNA target sequences are bound by Hfq as described in our recent study on the Hfq–Crc–*amiE*$_{6ARN}$ assembly (Pei *et al*, 2019), where the Hfq distal surface engages the ARN motifs as noted by Link *et al* (2009). In this interaction, the A- and R-site bases are bound in basic pockets on the Hfq distal side, while the N-bases are exposed, as such presenting the RNA for interaction with Crc partner molecules. Interestingly, all three complexes constitute a modular assembly that consists of one (for the *estA*$_{118}$ and *rbsB*$_{110}$ *assemblies*) or two (for the *amiE*$_{105}$ *assembly*) copies of the same Hfq–Crc–Crc core unit (Fig 4A–C), where a basic patch on the two Crc molecules engages mainly the phosphate backbone of the presented ARN motif (Fig 4C and D). To test the importance of this basic patch *in vivo*, translational *lacZ* reporter genes were designed for each target mRNA. The translation

 © 2022 The Authors

repression of these reporter constructs was then assessed in the presence of different Crc mutant proteins. Indeed, substitution of basic residues at the interface (Crc mutant R140E and quadruple mutant R140A/R141A, R138A/R139A) reduced translation repression to Δ*Crc* levels for all three reporter genes *amiE*+60::*lacZ*, *estA*+18::*lacZ*, and *rbsB*+13::*lacZ* (Fig 4H). These *in vivo* results corroborate the importance of the RNA interactions for translational repression of mRNA targets by Crc. For the Hfq–$amiE_{105}$–Crc assembly, the downstream $amiE_{xARN}$ motif in the proximal coding region (Fig 2A) only partially occupies the Hfq distal side yet still recruits 2 Crc molecules (Crc1 and 4; Hfq1 in Figs EV3A and 4A and B, left panels). Similarly, incomplete ARN-rich motifs partially decorate the Hfq distal sides in the $estA_{118}$ and $rbsB_{110}$ complexes (Fig EV3B and C), recruiting one or two additional Crc molecules per Hfq hexamer to the assembly (Fig 4B). All Crc molecules bind to the presented ARN motifs *via* the basic patch described above.

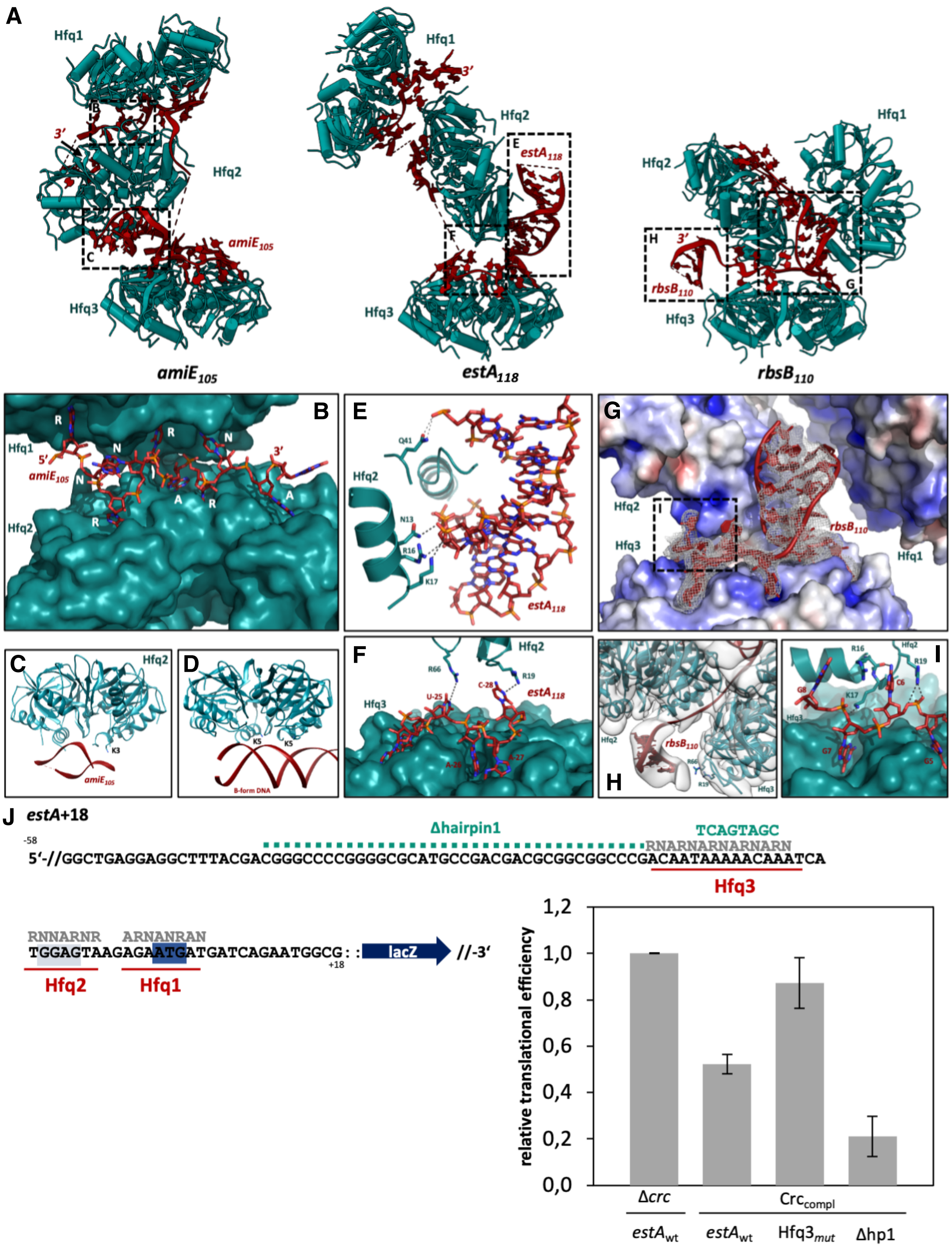

Figure 5.

Figure 5. RNA sequence motifs and secondary structure elements drive oligomerization of the translation repression complexes.

A Hfq–RNA components of the repressive complexes formed on *amiE*$_{105}$, *estA*$_{118}$, and *rbsB*$_{110}$ RNAs, shown without Crc for clarity.

B Close-up of the second ARN-rich motif of *amiE*$_{105}$, which is shared between the Hfq1 and Hfq2 distal sides. The R-site bases in the ARN repeats occupy alternating R-site pockets on the two Hfq distal sides, enabling higher order assembly formation.

C Close-up of a hairpin structure in *amiE*$_{105}$, which is coordinated by the Hfq2 proximal side (see also extended view Fig 3A, hairpin 1). Lys3 on the proximal helix of a Hfq 2 protomer is likely to form a polar contact with the RNA backbone in the duplex.

D Crystal structure of *Escherichia coli* Hfq bound to B-form DNA (Orans *et al*, 2020), where the DNA duplex binds the proximal face of the Hfq hexamer, analogous to the *amiE*$_{105}$ hairpin in (C).

E Close-up of a hairpin structure in *estA*$_{118}$, which is coordinated by the Hfq2 proximal side (see also extended view Fig 3B, hairpin1). Asn 13, Arg16, and Lys17 on the Hfq proximal helix coordinate the RNA backbone, as well as Gln41 on a proximal loop.

F The ARN motif is bound to the Hfq3 distal side in the *estA*$_{118}$ assembly is shared with the Hfq2 rim side. In particular, Arg19 and Arg66 form hydrogen bonds with the C-28 and U-25 bases, respectively.

G A duplex-like RNA fold is coordinated by basic patches on the Hfq2 and Hfq3 distal and proximal surfaces, respectively, in the *rbsB*$_{110}$ repressive complex.

H At low threshold, density for an RNA duplex at the *rbsB*$_{110}$ 3′-end is apparent (see also extended Fig 2C hairpin1) and is coordinated by the Hfq2 proximal side. The Hfq3 rim side can make putative hydrogen bonds with the hairpin backbone via Arg66 and Arg19.

I As in (B) and (F), a short ARN motif in the *rbsB*$_{110}$ sequence is shared between the Hfq3 distal side and the Hfq2 rim/proximal side. Hfq2 Arg16, Lys17, and Arg19 form contacts with the presented RNA backbone and bases in *rbsB*$_{110}$. In all three complexes, these Hfq–RNA–Hfq contacts drive higher order assembly formation.

J Translational regulation of the *estA*+18::*lacZ* reporter gene and mutants thereof by Hfq and Crc. The mRNA sequence of the 5′UTR and the proximal coding region of *estA*+18::*lacZ*. ARN motifs bound to Hfq distal sides are annotated. The Shine–Dalgarno sequence is highlighted in gray, and the AUG start codon is highlighted in blue. Mutations in the RNA sequence are indicated in green (sequence changes in the Hfq3-binding RNA segment and removal of the hairpin region). ARN-rich recognition sites for Hfq1, Hfq2 and Hfq3 are annotated in gray above the sequence. When compared with strain PAO1Δcrc (pME4510crc$_{Flag}$, p*estA*+18::*lacZ*; *estA* wt), loss of the Hfq3 (Hfq3mut) binding element diminished the repressive effects on estA mRNA significantly in strain PAO1Δcrc (pME4510crc$_{Flag}$, pestA+13Δ*CA*:::*lacZ*), whereas removal of the hairpin structure (Δhp1) results in a slight increase in translation repression in strain PAO1Δcrc (pME4510crc$_{Flag}$, pestA++18-Δhp1::*lacZ*). The β-galactosidase activities were normalized to the respective mRNA levels (= relative translational efficiency) to account for intrinsic changes in mRNA stabilities eventually arising from the mutations introduced into the *estA* segment.

Together, the structures of the translation repression assemblies and the validation studies *in vivo* indicate the importance of the common RNA interactions by Crc.

In the *amiE*$_{105}$ and the *rbsB*$_{110}$ repressive assemblies, Crc forms homodimers, and three different self-dimerization interfaces were observed (labeled as 1, 2, and 3 in Fig 4E–G), constituting a second recurring feature in the repressive assemblies. Since Crc is monomeric in solution at high μM concentrations, these dimerization interfaces must require that the Crc molecules are organized in repressive assemblies. Substitution of Glu193 for Arg is predicted to disrupt the Crc interfaces 2 and 3 in the *amiE*$_{105}$ and *rbsB*$_{110}$ assemblies, respectively, due to electrostatic repulsion and impact translation repression (Fig 4G). Indeed, the E193R substitution in Crc reduced the translation repression of the *amiE*+60::*lacZ* and *rbsB*+13::*lacZ* reporter genes to Δ*Crc* levels (Fig 4H). Compensation of the repulsive Crc interface by the R230E substitution in Crc in turn restored translation repression for the same *amiE* and *rbsB* reporter genes (Fig 4H). Although the Crc–Crc interfaces 2 and 3 are absent in the *estA*$_{118}$ complex, Crc residue E193 provides an alternative interaction with Hfq2 N28 (chain V) and Hfq2 R19 (chain V) that is anticipated to be weakened by the E193R substitution, which might account for the observed reduced repression effect for the *estA*+18::*lacZ* reporter gene as well (Fig 4H). The substitution R230E in Crc can form interactions with Hfq2 N28 and R19 of Hfq (chain V) and can restore the Hfq/Crc interface to compensate for the E193R substitution in the *estA*$_{118}$ complex. Lastly, the hydrogen-bonding interactions of R90 and Y91 in interface 3 of the *rbsB*$_{110}$ complex were tested with the double mutant R90E and Y91F, and found to have roughly a 2-fold effect on translational repression (Fig 4H). Interface 3 does not occur in either the *amiE*$_{105}$ or *estA*$_{118}$ complexes, where instead R90 and Y91 of Crc interact with the C-terminal tail of a Hfq protomer. The double mutation R90E and Y91F de-repressed translation of the *amiE* and *estA* reporter genes roughly 7-fold and 2-fold, respectively. In summary, these results show that the Crc interaction surfaces can be directed to either form self-complementary contacts that support Crc–Crc interactions or contact Hfq, both of which stabilize the polymorphic higher order repressive assemblies.

A third salient feature of the complexes is how the RNA is shared between adjacent Hfq molecules, where some Hfq distal sides present ARN motifs to the distal face or the rim of a neighboring Hfq hexamer, rather than to Crc partner molecules. In the Hfq–*amiE*$_{105}$–Crc assembly, the second *amiE*$_{xARN}$ motif in the *amiE* coding region is partially shared between the distal faces of Hfq1 and Hfq2, with nucleobases occupying alternating R-site pockets on both distal sides (Figs 5A and B, and EV3A). This sharing of *amiE*$_{xARN}$ in the *amiE* coding region between Hfq molecules drives higher order assembly formation and efficiently masks the ribosome-binding site, rationalizing the observation that the downstream ARN cluster enhances translational repression of *amiE in vivo* (Fig 2C and D). In the Hfq–*estA*$_{118}$–Crc complex, Hfq3 presents the first longer 5′ ARN motif (Fig EV3B, 12-mer, four ARN triplet repeats) to the Hfq2 rim side (Fig 5F). The Hfq2 rim side residues Arg19 and Arg66 form hydrogen bonds with the exposed N-site bases C-28 and U-25 of *estA*$_{118}$ (counting backward from the AUG start codon, with A annotated as nucleotide 1). Indeed, disruption of the ARN pattern of this Hfq3-binding site in the *estA* sequence resulted in a decrease in translation repression of the corresponding reporter gene by an order of magnitude *in vivo* (Fig 5J, Hfq3$_{mut}$). Such disruption would also abrogate binding of *estA* by the Hfq3 distal side. A similar yet more extensive Hfq-to-Hfq presentation of the RNA target is found in the Hfq–*rbsB*$_{110}$–Crc assembly, where a short RNRN motif in the *rbsB*$_{110}$ coding region is presented by the Hfq3 distal side to the Hfq2 rim/proximal side (Fig 5I). In particular, Hfq2 residues Arg16, Lys17, and Arg19 form hydrogen bonds with the phosphate backbone and the C6 nucleobase of *rbsB*$_{110}$ (counting from the AUG start codon, with A annotated as nucleotide 1). From these observations, it is apparent that completion of higher order assembly enhances translational repression, and that RNA-mediated Hfq–Hfq oligomerization drives this process.

Finally, in all three complexes, RNA duplex elements interact with the Hfq proximal sides in a sequence independent manner

 © 2022 The Authors

(Figs 5A, C, E, G, and H, and EV3). Although the local resolutions were not sufficient to resolve side chains, it is apparent that these interactions are between basic and polar residues in the proximal Hfq α-helix and the phosphate backbone of the RNA duplex structures (Fig 5C and E). In particular, the Hfq residues Lys3, Asn13, Arg16, and Lys17 are likely candidates for such interactions (Fig 5C and E). This mode of RNA secondary structure recognition by Hfq is in line with findings of a recent crystallographic study by Orans *et al* (2020), where *E. coli* Hfq was observed to interact with the phosphate backbone of B-form DNA (Fig 5D). It is unclear what role is played by this common mode of RNA duplex binding by Hfq in the repressive assemblies. For $estA_{118}$, for example, removing the hairpin structure from the RNA construct resulted in a significantly stronger translation repression of the *estA*+18::*lacZ* reporter gene *in vivo* (Fig 5J). Thus, the structural elements might confer either stabilizing or destabilizing contributions, depending on context.

In summary, the models of the repressive complexes formed on the $amiE_{105}$, $estA_{118}$, and $rbsB_{110}$ transcripts and the *in vivo* reporter gene assays support a model in which the sharing of the target RNA between Hfq/Crc molecules drives formation of higher order assemblies that fully mask the ribosome-binding sites. Ultimately, the RNA sequence determines the quaternary structure of such translation repression complex, through blocks of ARN repeats that can have different spacing and local imperfections (Fig EV3), and through secondary structure elements. The polymorphic character of the assembly in turn is accommodated by the hexameric character of Hfq, presenting a mosaic of basic patches on its surface, and consolidating interactions mediated through the permissive dimerization of organized Crc molecules. The different possible translation-repression assemblies, however, are all likely to fold following the recurring architectural principles described above.

Discussion

In *P. aeruginosa*, CCR controls not only carbon metabolism but also other complex behavior including infection, virulence, biofilm formation, and quorum sensing. We have shown here that a key component of CCR, namely the RNA chaperone Hfq, can form higher order assemblies on target mRNAs in conjunction with the protein Crc, and that such assemblies repress translation. Our cryo-EM analyses reveal distinct quaternary organizations of the assemblies on the regulatory regions of the *amiE*, *estA*, and *rbsB* mRNAs, which encode metabolic and virulence machinery, and are known to be down-regulated during CCR (Sonnleitner & Bläsi, 2014; Kambara *et al*, 2018).

From our cryo-EM structures, we have elaborated rules that encode the architectural principles of translation repression assemblies based on four recurring features of the complexes. Firstly, the distal face of Hfq hexamer engages ARN-rich repeats, which is the sequence specificity determining factor. Crc can then interact with these elements *via* a distinct basic patch on its surface. As such, the complexes are characterized by a common core sub-assembly, comprised of one Hfq hexamer which presents an ARN-rich motif to two Crc molecules (Fig 4B and C). The second recurring feature is that the proximal side of Hfq binds secondary structure elements in the RNA targets. There are some puzzling aspects of RNA duplex binding by Hfq in the repressive assemblies. For $estA_{118}$, for example, removing the hairpin structure from the RNA construct resulted in a significantly stronger translation repression of the *estA*+18::*lacZ* reporter gene *in vivo* (Fig 5J), while stabilizing a potential stem-loop structure in the coding region of *amiE* also increased translational repression. RNA structure impacts on Hfq binding (Hopkins *et al*, 2009; Ishikawa *et al*, 2012), and the effects of these secondary structures in the RNA might compete with or contribute to the formation of the higher order assemblies, depending on the context of the full assembly. Thirdly, higher order folding is driven by sharing of RNA segments between Hfq protomers and is enabled by the hexameric ring organization of Hfq, in which protomers rich in RNA-binding patches provide repetitive RNA interaction sites. Notably, the Hfq–Hfq and Hfq–Crc interfaces formed in the translation repression complexes are almost exclusively through mutual interactions with the RNA substrate. In other words, Hfq and Crc need to bind to a polymer (RNA) for higher order assemblies to form. Polymer-bound proteins have a higher propensity to interact with each other, as part of the entropy is already lost, enabling small surfaces to contribute. As the fourth rule, we note that some of these small surfaces are formed between neighboring Crc molecules in the complex. These Crc dimerization interfaces support the distinct quaternary structures, and its surfaces can switch between self-complementary association or interaction with RNA/Hfq. From these principles, a diversity of quaternary structures can be supported that can regulate numerous genes in *P. aeruginosa* with minimal proteinogenic components required.

Hfq is highly pleiotropic and involved in many riboregulatory processes. The envisaged multi-faceted control of CCR and processes linked to it entails a hidden cost of apparently requiring high numbers of Hfq and Crc in the cell, and the question arises if levels of the chaperone available are sufficient to meet the demands of forming higher order assemblies. Given that each mRNA leads to 100–1,000 translated metabolic proteins on average, translational repression is only energetically beneficial for the cell if it is effective (Lynch & Marinov, 2015). Hfq levels in *P. aeruginosa* have been calculated at ~ 2,200 hexamers per cell during stationary phase, and nearly equimolar Crc levels per cell (~ 2,300) were measured (Sonnleitner & Bläsi, 2014; Sonnleitner *et al*, 2018). One scenario is that the pool is highly dynamic, and that Hfq and Crc become available through rapid turnover of transcripts to which they are bound. Indeed, transcripts are poised for degradation after translational repression (Sonnleitner & Bläsi, 2014), likely releasing Hfq and Crc molecules that were sequestered in the repressive assembly. We envisage that the assemblies would be accessible for delivery to and action by the RNA decay machinery in analogy to the processes studied for untranslated RNA (Iost & Dreyfus, 1995). Also, genes encoding degradative proteins involved in metabolic pathways are generally only transcribed if the respective carbon source is available. This means that only a limited number of genes need to be translationally repressed in a given nutrient environment, i.e., the genes involved in metabolism of available yet non-favorable carbon sources. Lastly, it is possible that repressive responses might be graded through partial assemblies. Thus, sub-assemblies, such as the Hfq–Crc–Crc core unit, might yield meaningful translation repression even when Hfq/Crc levels are insufficient to drive higher order ribonucleoprotein folding. For instance, our *in vivo* studies indicate that the downstream $amiE_{xARN}$ motif of *amiE* present in the coding region ensures a more complete repression, but the absence

of this secondary motif still results in significant translation repression (Fig 2D).

The biological impact of these assemblies might depend on windows of opportunity arising during the synthesis of the transcript. Sequential assembly of the Hfq–Crc complexes is envisaged to occur on nascent transcripts as they emerge from the RNA polymerase, potentially coupled with RNA folding in analogy with other systems (Kambara *et al*, 2018; Yu *et al*, 2021). Co-transcriptional folding of the RNA, for instance, the formation of stem-loop structures that influence the quaternary architectures, could affect the rates of assembly and stabilities of the complexes. Single-molecule studies have shown that target RNA secondary structure presents a kinetic energy barrier that determines whether target recognition occurs before stable pairing to sRNA (Malecka & Woodson, 2021). Extending this principle, we envisage that critical kinetic steps also occur in the assembly of Hfq–Crc complexes on nascent transcripts. Indeed, removing the prominent hairpin structure that is recognized by the central Hfq in the $estA_{118}$ repressive complex (Fig 5A and E, Hfq2) promotes translational repression *in vivo*, suggesting a somewhat antagonistic role for such hairpin structures in the assembly pathway, as discussed above. Co-transcriptional Hfq–Crc assembly on an RNA target might bear analogy to the synergistic co-transcriptional assembly of ribosomes on rRNA (Rodgers & Woodson, 2019). If so, stepwise Hfq–Crc assembly is not sequential, but rather depends on the contextual state of the RNA-binding sites and the presence of other copies of Hfq/Crc proteins. The difference here is that Hfq/Crc assembly is tuned temporally, and the resulting translation repression complexes are transient in nature, subject to kinetic competition, as distinct from folding equilibrium complexes such as ribosomes and spliceosome components (Herzel *et al*, 2017; Rodgers & Woodson, 2019). Another indication that assembly of repressive complexes might be coupled to the transcription machinery is the observation that the 3′-end of transcripts that are repressed during CCR are diminished up to 10-fold in RNA sequencing analyses (Sonnleitner *et al*, 2018). One mechanism that could explain this observation is the recruitment of transcription termination factors during formation of the Hfq/Crc complexes, coupling translation-repression of a transcript to termination of its transcription. This hypothesis awaits validation.

The response to environmental changes and stress, and the rerouting of metabolic pathways demand systems of hierarchical control that form highly inter-connected networks. Such an intricate system is a demanding process for the cell, requiring many specificity factors, i.e., protein components, to function properly. Here, we observe that specificity can be achieved with only two multifaceted protein factors and patterns in the RNA sequence with a weak yet distinct grammar. The modular nature of the protein factors and their mode of RNA interaction enable different quaternary organizations to result, i.e., less stringent folding requirements for assembly. The regulatory outcome then becomes dependent on competitive kinetics of Hfq–Crc assembly versus translational initiation, i.e., binding of the 30S small ribosomal subunit to the mRNA ribosome-binding site. Such an economic design also permits rapid modifications in network organization in the course of evolution through changes in the patterns of target RNA elements. In summary, polymorphism of folded ribonucleoprotein complexes allows for a simple, highly modular regulon that underpins the noted behavioral complexity of *P. aeruginosa*.

Materials and Methods

Pseudomonas aeruginosa Hfq purification

An Hfq-deficient *Escherichia coli* strain bearing plasmid pKEHfqPae encoding *Pseudomonas aeruginosa* Hfq (Sonnleitner *et al*, 2018) was grown in 50 ml Lysogeny broth (LB; Miller, 1972) supplemented with 0.2% wt/v glucose, 15 μg/ml kanamycin, and 50 μg/ml ampicillin at 37°C in an orbital shaker at 220 rpm overnight. A 5 ml of the pre-culture was used to inoculate 4 L of LB (with the same supplements) at 37°C, and the culture was grown in a shaker to an OD_{600nm} of 0.6, at which time expression of the *hfq* gene was induced with 1 mM IPTG (isopropyl β-D-1-thiogalactopyranoside). After continued growth at 4 h, the cells were harvested by centrifugation at 5,000 *g* for 20 min at 4°C, and the pellets were resuspended in 20 ml lysis buffer (50 mM Tris–HCl pH 8, 1.5 M NaCl, 250 mM $MgCl_2$, 1 mM β-mercaptoethanol, 1 mM EDTA, 1 mM PMSF) and frozen in liquid nitrogen for storage at −80°C. The thawed cells were supplemented with 20 μg/ml DNase I and lysed using an Avestin Emulsiflex C5 homogenizer (5 passes, 1,000 bar). The lysate was centrifuged at 35,000 *g* for 20 min at 4°C, and the supernatant was collected and heated to 85°C in a water bath for 45 min. The precipitate was removed by a 20,000 *g* spin at 4°C for 15 min and 1 M $(NH_4)_2SO_4$ was gradually added to the supernatant. The precipitate was pelleted at 20,000 *g* for 15 min (4°C), and then the supernatant was filtered through a 0.42 μm Sartorius Minisart syringe filter. The sample was applied to a 5 ml HiTrap Butyl HP column (GE Lifesciences) equilibrated in buffer A (50 mM Tris–HCl pH 8, 1.5 M NaCl, 1.5 M $(NH_4)_2SO_4$, 0.5 mM β-mercaptoethanol, 0.5 mM EDTA, and 0.1 mM PMSF). After loading, 10 column volumes of buffer A were used to remove contaminants, and a 0–100% linear gradient of buffer B (50 mM Tris–HCl pH 8.0, 200 mM NaCl, 0.5 mM β-mercaptoethanol, 0.5 mM EDTA, and 0.1 mM PMSF (phenylmethylsulfonyl fluoride)) was used to elute Hfq. The eluted protein was diluted 2-fold in buffer Hep-A (50 mM Tris–HCl pH 8.0, 100 mM NaCl, 0.5 mM β-mercaptoethanol, 0.5 mM EDTA, and 0.1 mM PMSF) and loaded on a 5 ml HiTrap Heparin column (GE Lifesciences) equilibrated with buffer Hep-A. About 10 column volumes of Buffer Hep-A were then used to wash of any remaining contaminants. Hfq was eluted with a linear 0–60% gradient of buffer Hep-B (50 mM Tris–HCl pH 8.0, 2 M NaCl). Next, the peak fractions were pooled and concentrated in an Amicon Ultra centrifugal filter unit (10 kDa cutoff) to a final volume of 500 μl. The sample was then loaded on a Superdex 200 Increase 10/300 GL (GE Lifesciences) equilibrated with Buffer SEC-A (50 mM Tris–HCl pH 7.5, 200 mM NaCl, 10% v/v glycerol). The peak fractions were flash frozen and stored at −80°C. An SDS–PAGE denaturing gel was run with the peak fractions to assess purity.

Pseudomonas aeruginosa Crc purification

Plasmid pETM14lic-6His-Crc was transformed into competent *E. coli* BL21DE3 cells by standard heat shock transformation (Milojevic *et al*, 2013), and the cells were plated on LB-agar plates supplemented with 50 μg/ml kanamycin. A pre-culture of the cells was grown overnight in 50 ml LB medium supplemented with 50 μg/ml kanamycin. A 4 × 800 ml of LB, supplemented with 50 μg/ml kanamycin, 0.2% glucose, and 2 mM $MgSO_4$ were inoculated with 4 ml

 © 2022 The Authors

of the pre-culture. A 3 mM of IPTG (final concentration) was used to induce expression of the crc gene at an OD_{600nm} of 0.6. After 3 h, the cells were harvested by centrifugation at 5,000 *g* for 20 min. The pellet was resuspended in 50 ml Ni-A buffer (50 mM Tris–HCl pH 8.0, 300 mM NaCl, 10 mM imidazole, 1 mM β-mercaptoethanol, 0.1 mM PMSF), frozen in liquid nitrogen, and stored at −80°C. The thawed cells were supplemented with 20 μg/ml DNase I and 20 μg/ml RNase A and lysed using an Avestin Emulsiflex C5 homogenizer (5 passes, 1,000 bar). The lysate was centrifuged for 30 min at 30,000 *g* (4°C), and the supernatant was loaded on a 5 ml HiTrap chelating column charged with $NiSO_4$ and equilibrated in buffer Ni-A. The column was washed with 10 column volumes of buffer Ni-W (50 mM NaH_2PO_4, 300 mM NaCl, 20 mM imidazole, pH 8.0) and eluted with a linear 0–60% gradient of buffer Ni-B (50 mM NaH_2PO_4, 300 mM NaCl, 500 mM imidazole, pH 8.0). The peak fractions were pooled and dialyzed in 50 mM Hepes pH 8, 150 mM NaCl, and 1 mM β-mercaptoethanol, and the concentration was measured with a NanoDrop spectrophotometer (Thermo Fisher). For each milligram of protein, 20 μg PreScission Protease (Sigma Aldrich) was added to cleave the His-tag. After 2 h of incubation at 4°C, the sample was applied to a nickel column to remove the cleaved His-tags and PreScission Protease, and the flow through was concentrated with an Amicon Ultra centrifugal filter (5 kDa molecular weight cutoff). The sample was loaded on a Superdex 200 Increase 10/300 GL (GE Lifesciences) equilibrated with Buffer SEC-A (50 mM Hepes pH 8.0, 150 mM NaCl, 1 mM TCEP (tris(2-carboxyethyl) phosphine) and 10% v/v glycerol). The peak fractions were flash frozen and stored at −80°C. An SDS–PAGE denaturing gel was run with the peak fractions to assess purity.

Bacterial strains/plasmids used in this study and construction of *lacZ*:: reporter genes

To construct translational gene fusions between *amiE* and *lacZ*, DNA fragments containing nucleotides from −242 to +3 (*amiE*+3) and to +60 (*amiE*+60), respectively, with regard to the A (+1) of the start codon of *amiE* were amplified by PCR using the oligonucleotides A1 (5′-TTTTTTGAATTCGGCTGCATGCTATCTCAGGCGC-3′) and either F173 (*amiE*+3; 5′-TTTTTTCTGCAGGTAGTTGACCACCGCCACTC-3′) or G173 (*amiE*+60; 5′-TTTTTTCTGCAGCATGGATATCACCTCTTGTTG-3′) and chromosomal DNA of strain PAO1 (Holloway *et al*, 1979) as template. The PCR fragments were cleaved with EcoRI and PstI and then ligated into the corresponding sites of plasmid pME6015 (Schnider-Keel *et al*, 2000), generating plasmids pamiE+3::lacZ and pamiE+60::lacZ, respectively. Plasmid prbsB+13::lacZ was constructed as described in Kambara *et al* (2018). A DNA fragment containing nucleotides from −372 to +13 with regard to the A (+1) of the start codon of *rbsB* was amplified by PCR using the oligonucleotide pair T191 (5′-ATATGAATTCGTCCAGCCTGGAGGTCTACAAG-3′) and U191 (5′-ATATCTGCAGCGACCCGCTTCATGGTG-3′) and chromosomal DNA of strain PAO1 as template. The PCR fragments were cleaved with EcoRI and PstI and then ligated into the corresponding sites of plasmid pME6015 (Schnider-Keel *et al*, 2000), generating plasmid prbsB+13::lacZ.

The construction of pestA+18::lacZ has been described by Sonnleitner & Bläsi (2014), wherein it was termed pTLestA. Plasmid pestA+18::lacZ contains a DNA fragment of *estA* spanning nucleotides −580 to +18 with regard to the A (+1) of the start codon of *estA*.

Plasmid pTLestA-ΔCA (herein termed pestA+13ΔCA::lacZ), wherein a part of the Hfq3-binding site (AAAACAA) in *estA* was mutated to TCAGTAGC ($Hfq3_{mut}$ in Fig 4), has been described in Huang *et al* (2012).

Plasmid pestA+18-Δhp1::lacZ was constructed employing overlapping PCR. The PCR fragments were amplified with primer pairs Q67 (5′-TTTTGAATTCGAGCAGCCTGGCACGC-3′)/G191 (5′-TCGTAAAGCCTCCTCAG-3′) and H191 (5′-CTGAGGAGGCTTTACGAACAATAAAAACAAATCATGGAGTAAGAGA-3′)/R67 (5′-TTTTGGATCCGAGCGCCATTCTGATCAT-3′) and pestA+18::lacZ as template. The PCR fragments were combined and used as a template for a second overlapping PCR with primers Q67 and R67. The resulting PCR fragment, comprising fragment of *estA* from nucleotide −580 to +18 with a deletion of 37 nucleotides from nucleotides −66 to −30 with regard to the A (+1) of the start codon of *estA*, was digested with EcoRI and BamHI and ligated into the corresponding sites of pME6015 (Schnider-Keel *et al*, 2000).

Construction of plasmids encoding Crc variant proteins

Derivatives of plasmid pME4510crc_{Flag} (Sonnleitner *et al*, 2018) were constructed by site-directed mutagenesis using plasmid pME4510crc_{Flag} as template and the mutagenic oligonucleotide pairs E191 (5′-GAGCAAGCAGCGTGCCGCGGCCGCCGAATACATCTACTGC-3′)/F191 (5′-GCAGTAGATGTATTCGGCGGCCGCGGCACGCTGCTTGCTC-3′; $Crc_{R138A,K139A,R140A,R141A}$), N191 (5′-CGATCGTTACGGGGAATTCCTGCAAGCCGACTTCGACAAGG-3′)/O191 (5′-CCTTGTCGAAGTCGGCTTGCAGGAATTCCCCGTAACGATCG-3′; $Crc_{R90E,Y91F}$), L191 (5′-CCTTTATGCCTGCGATGCCCGTCTACCCGAACAGG-3′)/M191 (5′-CCTGTTCGGGTAGACGGGCATCGCAGGCATAAAGG-3′; CrcE61R), and A191 (5′-CTTAGGTTTCCGCACGGCCGATC-3′)/B191 (5′-GATCGGCCGTGCGGAAACCTAAG-3′; CrcE86R), respectively. The parental plasmid templates were digested with DpnI and the mutated nicked circular strands were transformed into *E. coli* XL1-Blue, generating plasmid pME4510$crc_{(R138A,K139A,R140A,R141A)Flag}$, pME4510$crc_{(R90E,Y91F)Flag}$, and pME4510$crc_{(E61R,E86R)Flag}$.

The construction of plasmids pME4510$crc_{(E193R)Flag}$, pME4510$crc_{(R230E)Flag}$, pME4510$crc_{(E193R,R230E)Flag}$, pME4510$crc_{(E193A,R230E)Flag}$, pME4510$crc_{(R229A,R230E)Flag}$, and pME4510$crc_{(R140E)Flag}$ was done as described in Pei *et al* (2019).

Preparation and purification of *amiE*$_{105}$, *rbsB*$_{110}$, and *estA*$_{118}$ RNA fragments

The RNA fragments *amiE*$_{105}$ (comprises nt −45 to +60 of *amiE* mRNA with respect to the A (+1) of the start codon), *rbsB*$_{110}$ (comprises nt −75 to +33 of *rbsB* mRNA with respect to the A (+1) of the start codon plus two additional G-nucleotides at the 5′ end), and *estA*$_{118}$ (comprises nt −85 to +33 of *estA* mRNA with respect to the A (+1) of the start codon) were prepared by *in vitro* transcription using T7 RNA polymerase. The DNA templates were amplified by PCR using the oligonucleotide pairs R185 (5′-TCTAGACGTAATACGACTCACTATAGGGCCTTTTTTCGTCCCGAAAAAATAACAAC-3′) and Z172 (5′-GTAGTTGACCACCGCCACTC-3′; *amiE*$_{105}$), P163 (5′-AGATAATACGACTCACTATAGGAACGCAAACGTTTGCGTCTGGATAATCTCCT-3′) and Q163 (5′-AGCCAACAGGCGCCGGGAAGCGACCCGCTTCAT-3′; *rbsB*$_{110}$), and T163 (5′-AGATAATACGACTCACTATAGGCTGAGGAGGCTTTACGACGGGCCCCGGGG-3′) and

U163 (5′-cgctaccagtggcttgagcgccattctgatCAT-3′; *estA*$_{118}$). The corresponding forward primers R185, P163 and T163, respectively, contained the T7 promoter sequence (underlined). After *in vitro* transcription with T7 RNA polymerase, the RNA fragments were gel purified using 8% polyacrylamide-8M urea gels.

Electrophoretic mobility shift assays (EMSA)

For the EMSAs, 4 μM stocks of Hfq and Crc were prepared in binding buffer (20 mM Tris–HCl pH 8.0, 40 mM NaCl, 10 mM KCl, 1 mM $MgCl_2$), and a 2 μM stock of the RNAs was prepared in milliQ water (RNase free). The RNAs were incubated at 50°C for 3 min before the proteins were added. The 4% poly-acrylamide (PAA) gels were used to study complex formation (6.73 ml acrylamide:bis-acrylamide, 5 ml 10× TBE, 37.7 ml milliQ water, 500 μl 10% APS, and 50 μl TEMED). Hfq was titrated into a mixture of the RNAs (*amiE*$_{105}$, *rbsB*$_{110}$, and *estA*$_{118}$) at different ratios in the presence or absence of an excess of Crc. The RNA concentration was kept constant at 200 nM. After 15 min of incubation at 37°C, the samples were mixed with an equal volume of loading buffer (50% v/v glycerol, 50% v/v binding buffer, 5 mM DTT), prior to loading them onto the gel. The gels were run at 4°C in 1× TBE running buffer and stained with SYBR gold.

Cryo-EM sample preparation

For Hfq–Crc assembly on *amiE*$_{105}$, the RNA was annealed at 50°C for 3 min. Hfq and Crc were mixed at 1.6 and 0.8–8.8 μM, respectively, prior to addition of *amiE*$_{105}$ (800 nM final concentration). After incubation on ice for 1 h, the mixture was diluted 7-fold before loading onto grids. The Hfq–*rbsB*$_{110}$–Crc complex was prepared following a similar procedure. The *rbsB*$_{110}$ fragment was annealed at 50°C for 3 min. Hfq and Crc were mixed at 2.8 μM and 9 μM, respectively, after which the RNA was added at 400 nM. After incubation at room temperature for 15 min and on ice for 1 h, the mixture was diluted 4-fold prior to grid preparation. The Hfq–*estA*$_{118}$–Crc complex was prepared following the same procedure as for the rbsB$_{110}$ assembly, but the final sample was not diluted prior to grid preparation.

Grid preparation

Graphene oxide (GO) grids were prepared from Quantifoil R1.2/1.3 grids. A 2 mg/ml graphene oxide dispersion (Sigma Aldrich) was diluted 10-fold and spun down at 300 *g* for 30 s to remove large aggregates. The dispersion was then diluted 10-fold before applying 1 μl to glow-discharged grids (0.29 mbar, 15 mA, 2 min, Pelco Easiglow glow discharger). After drying out, the grids were stored in a grid box for 24–48 h prior to usage. A 3 μl of the sample was applied to the GO grids, and after 30 s of incubation, excess sample was blotted away and frozen in liquid ethane (blot force −4 to 0, blot time 3 s, Vitrobot markIV (Thermo Fischer)). The grids were screened on a 200 kV Talos Arctica (FEI; Cryo-EM facility, Department of Biochemistry, University of Cambridge), and the movies were recorded on a 300 kV Titan Krios (Thermo Fischer) with either a Falcon III (Thermo Fischer) or K3 (Gatan) direct electron detector (MRC-LMB and BioCem facility, Department of Biochemistry, University of Cambridge).

Single-particle analysis, model building, and refinement

All datasets were pre-processed with Warp (Tegunov & Cramer, 2019). Particle sets were optimized in CryoSparc (Punjani *et al*, 2017) *via* repetitive 2D classifications and heterogeneous refinements. Further extensive classifications in 2D were used to classify different assemblies observed on the grid for each of the mRNA targets. High-resolution maps were generated for the highest order assemblies with non-uniform refinement in cryoSPARC (Punjani *et al*, 2020) and global and per particle CTF refinements (Table EV1). The Hfq–2Crc–*amiE*$_{105}$ (147,000 particles, Fig 2A), 2Hfq–3Crc–*amiE*$_{105}$ (99,000 particles, Fig 2A), and 3Hfq–4Crc–*amiE*$_{105}$ (70,000 particles) assemblies were refined to 3.2 Å, 3.9 Å, and 3.6 Å, respectively. The Hfq–Crc–*estA*$_{118}$ map was reconstructed 4.5 Å after global refinements and 4.1 Å after local, masked refinements. The Hfq–Crc–*rbsB*$_{110}$ map was refined to 3.8 Å.

Crystal structures for *P. aeruginosa* Crc (PDB code 4JG3) and Hfq (PDB code 1U1T) were manually docked into the EM density map as rigid bodies in Chimera (Pettersen *et al*, 2004). The *amiE*$_{105}$, *estA*$_{118}$, and *rbsB*$_{110}$ sequences were manually built into the density using Coot (Emsley *et al*, 2010). Refmac and Phenix real-space refinement were used to iteratively refine the multi-subunit complexes, followed by manual corrections for Ramachandran and geometric outliers in Coot and ISOLDE guided by sharpened maps (Table EV1; Emsley *et al*, 2010; Murshudov *et al*, 2011; Afonine *et al*, 2012; Burnley *et al*, 2017; Jakobi *et al*, 2017; Croll, 2018; Ramírez-Aportela *et al*, 2020). Model quality was evaluated with MolProbity (Williams *et al*, 2017).

In vivo expression of the translational reporter genes

The ability of Hfq, Crc, and Crc mutant proteins to repress the translation of the *amiE*+60::*lacZ*, *rbsB*+13::*lacZ*, and *estA*+18::*lacZ* reporter genes was tested in the *P. aeruginosa* strains PAO1 (Holloway *et al*, 1979), PAO1Δ*hfq* (Sonnleitner *et al*, 2018), and PAO1Δ*crc* (Sonnleitner *et al*, 2009) bearing plasmids pME4510 (vector control; Rist & Kertesz, 1998), pME4510crc$_{Flag}$ (encodes the *crc* wt gene; Sonnleitner *et al*, 2018), or derivatives thereof encoding the Crc mutant proteins described in the text. The strains were grown to an OD_{600} of 2.0 in BSM medium (Sonnleitner *et al*, 2009) supplemented with 40 mM succinate and 40 mM acetamide (*amiE*+60::*lacZ* fusions) or only 40 mM succinate (*rbsB*+13::*lacZ* and *estA*+18::*lacZ* fusions). The β-galactosidase activities were determined as described (Miller, 1972) using cells permeabilized with 5% toluene. The β-galactosidase units in the different experiments were derived from two independent experiments.

Determination of the relative translational efficiencies of the different *estA*+18::*lacZ* genes

To account for possible differences in mRNA stability of the different *estA*+18::*lacZ* mRNAs (Fig 4J), the relative translational efficiencies were determined by normalizing the β-galactosidase values to the mRNA levels assessed by qRT–PCR. First, total RNA was purified by the hot phenol method (Leoni *et al*, 1996). The remaining DNA was digested with Turbo DNase (Thermo Fisher Scientific). A 2 μg of total RNA was used for cDNA synthesis with AMV reverse Transcriptase (Promega) together with 20 pmol of oligonucleotides O135 (5′-TAGCGGCTGATGTTGAACTG-3′, binds to *lacZ*) and M37

 © 2022 The Authors

(5′-AGTCATGAATCACTCCGTGGTA-3′; binds to 16S rRNA). A 5 μl of 20-fold (*lacZ*) and 1,000-fold (16S rRNA) diluted cDNA samples, respectively, were used as templates for qPCR with HOT FIREpol® EvaGreen®qPCR mix (Solis biodyne) and 5 pmol of oligonucleotides (*lacZ*: O135/N135 (5′-ACTATCCCGACCGCCTTACT-3′); 16SrRNA: M37/L37 (5′-ATCGTAGTCCGGATCGCAGT-3′)) in a 20 μl reaction. The qPCR reaction was performed in a Realplex 2 Mastercylcer (Eppendorf). The PCR efficiencies and relative expression ratios of the target genes (*estA*+18::*lacZ* and mutants thereof) in comparison to the reference gene (16S rRNA) were calculated as described in Pfaffl (2001). The relative translational efficiencies were determined by normalizing the β-galactosidase values to the mRNA levels of the corresponding fusion genes and setting the relative translational efficiency of *estA*+18::*lacZ* in the absence of Crc to 1.

Data availability

The models and maps have been deposited with the PDB and EMDB, with PDB entries 8BVH, 8BVM, and 8BVJ, and corresponding EMDB entries ID EMD-16264, EMD-16266, and EMD-16265 for the *amiE*, *rbsB*, and *estA* complexes, respectively.

Expanded View for this article is available online.

Acknowledgments
The work was supported by a Wellcome Trust Investigator award to BFL (200873/Z/16/Z). TD was supported by an AstraZeneca studentship. UB and ES were supported by the Austrian Science Fund (FWF; FundRef Doi: 10.13039/501100002428; P28711-B22). We express our gratitude to Flavia Bassani and Armin Resch for initial experiments and the provision of materials. We thank Kasia Bandyra, Ewelina Malecka-Grajek, Nancy Standart, Giulia Paris, and Alexander Borodavka for helpful discussions and advice. All grids were prepared and cryo-EM data collected at the BIOCEM facility, Department of Biochemistry, University of Cambridge. We thank Dimitri Y. Chirgadze, Steven Hardwick, and Lee Cooper for assistance with data collection at the Cryo-EM Facility.

Author contributions
Ben F Luisi: Conceptualization; supervision; funding acquisition; validation; investigation; writing—original draft; project administration; writing—review and editing. **Tom Dendooven:** Conceptualization; formal analysis; investigation; writing—original draft; writing—review and editing. **Elisabeth Sonnleitner:** Formal analysis; investigation; methodology; writing—original draft; writing—review and editing. **Udo Bläsi:** Conceptualization; formal analysis; supervision; funding acquisition; investigation; writing—original draft; project administration; writing—review and editing.

Disclosure and competing interests statement
The authors declare that they have no conflict of interest.

References

Abdou L, Chou HT, Haas D, Lu CD (2011) Promoter recognition and activation by the global response regulator CbrB in *Pseudomonas aeruginosa*. *J Bacteriol* 193: 2784 – 2792

Afonine PV, Grosse-Kunstleve RW, Echols N, Headd JJ, Moriarty NW, Mustyakimov M, Terwilliger TC, Urzhumtsev A, Zwart PH, Adams PD (2012) Towards automated crystallographic structure refinement with phenix.refine. *Acta Crystallogr D Biol Crystallogr* 68: 352 – 367

Andrade JM, dos Santos RF, Chelysheva I, Ignatova Z, Arraiano CM (2018) The RNA-binding protein Hfq is important for ribosome biogenesis and affects translation fidelity. *EMBO J* 37: e97631

Burnley T, Palmer CM, Winn M (2017) Recent developments in the CCP-EM software suite. *Acta Crystallogr D Struct Biol* 73: 469 – 477

Chen J, Gottesman S (2017) Hfq links translation repression to stress-induced mutagenesis in *E. coli*. *Genes Dev* 31: 1382 – 1395

Corona F, Reales-Calderon JA, Gil C, Martinez JL (2018) The development of a new parameter for tracking post-transcriptional regulation allows the detailed map of the *Pseudomonas aeruginosa* Crc regulon. *Sci Rep* 8: 16793

Croll TI (2018) ISOLDE: a physically realistic environment for model building into low-resolution electron-density maps. *Acta Crystallogr D Struct Biol* 74: 519 – 530

Dendooven T, Sinha D, Roeselová A, Cameron TA, De Lay NR, Luisi BF, Bandyra KJ (2021) A cooperative PNPase-Hfq-RNA carrier complex facilitates bacterial riboregulation. *Mol Cell* 81: 2901 – 2913

dos Santos RF, Arraiano CM, Andrade JM (2019) New molecular interactions broaden the functions of the RNA chaperone Hfq. *Curr Genet* 65: 1313 – 1319

Dubey AK, Baker CS, Romeo T, Babitzke P (2005) RNA sequence and secondary structure participate in high-affinity CsrA-RNA interaction. *RNA* 11: 1579 – 1587

Emsley P, Lohkamp B, Scott WG, Cowtan K (2010) Features and development of Coot. *Acta Crystallogr D Biol Crystallogr* 66: 486 – 501

Fernandez L, Breidenstein EB, Taylor PK, Bains M, de la Fuente-Nunez C, Fang Y, Foster LJ, Hancock RE (2016) Interconnection of post-transcriptional regulation: the RNA-binding protein Hfq is a novel target of the Lon protease in *Pseudomonas aeruginosa*. *Sci Rep* 6: 26811

Gebhardt MJ, Kambara TK, Ramsey KM, Dove SL (2020) Widespread targeting of nascent transcripts by RsmA in *Pseudomonas aeruginosa*. *Proc Natl Acad Sci U S A* 117: 10520 – 10529

Goodman AL, Merighi M, Hyodo M, Ventre I, Filloux A, Lory S (2009) Direct interaction between sensor kinase proteins mediates acute and chronic disease phenotypes in a bacterial pathogen. *Genes Dev* 23: 249 – 259

Herzel L, Ottoz DSM, Alpert T, Neugebauer KM (2017) Splicing and transcription touch base: co-transcriptional spliceosome assembly and function. *Nat Rev Mol Cell Biol* 18: 637 – 650

Holloway BW, Krishnapillai V, Morgan AF (1979) Chromosomal genetics of Pseudomonas. *Microbiol Rev* 43: 73 – 102

Holmqvist E, Wright PR, Li L, Bischler T, Barquist L, Reinhardt R, Backofen R, Vogel J (2016) Global RNA recognition patterns of post-transcriptional regulators Hfq and CsrA revealed by UV crosslinking *in vivo*. *EMBO J* 35: 991 – 1011

Hopkins JF, Panja S, McNeil SAN, Woodson SA (2009) Effect of salt and RNA structure on annealing and strand displacement by Hfq. *Nucleic Acids Res* 37: 6205 – 6213

Huang J, Sonnleitner E, Ren B, Xu Y, Haas D (2012) Catabolite repression control of pyocyanin biosynthesis at an intersection of primary and secondary metabolism in *Pseudomonas aeruginosa*. *Appl Environ Microbiol* 78: 5016 – 5020

Iost I, Dreyfus M (1995) The stability of Escherichia coli lacZ mRNA depends upon the simultaneity of its synthesis and translation. *EMBO J* 14: 3252 – 3261

Ishikawa H, Otaka H, Maki K, Morita T, Aiba H (2012) The functional Hfq-binding module of bacterial sRNAs consists of a double or single hairpin preceded by a U-rich sequence and followed by a 3′ poly(U) tail. *RNA* 18: 1062 – 1074

© 2022 The Authors

Jakobi AJ, Wilmanns M, Sachse C (2017) Model-based local density sharpening of cryo-EM maps. *Elife* 6: e27131

Kambara TK, Ramsey KM, Dove SL (2018) Pervasive targeting of nascent transcripts by Hfq. *Cell Rep* 23: 1543–1552

Krepl M, Dendooven T, Luisi BF, Sponer J (2021) MD simulations reveal the basis for dynamic assembly of Hfq-RNA complexes. *J Biol Chem* 296: 100656

Leoni L, Ciervo A, Orsi N, Visca P (1996) Iron-regulated transcription of the *pvdA* gene in *Pseudomonas aeruginosa*: effect of Fur and PvdS on promoter activity. *J Bacteriol* 178: 2299–2313

Link TM, Valentin-Hansen P, Brennan RG (2009) Structure of *Escherichia coli* Hfq bound to polyriboadenylate RNA. *Proc Natl Acad Sci USA* 106: 19292–19297

Lu P, Wang Y, Zhang Y, Hu Y, Thompson KM, Chen S (2016) RpoS-dependent sRNA RgsA regulates Fis and AcpP in *Pseudomonas aeruginosa*. *Mol Microbiol* 102: 244–259

Lynch M, Marinov GK (2015) The bioenergetic costs of a gene. *Proc Natl Acad Sci U S A* 112: 15690–15695

Malecka EM, Bassani F, Dendooven T, Sonnleitner E, Rozner M, Albanese TG, Resch A, Luisi BF, Woodson S, Bläsi U (2021) Stabilization of Hfq-mediated translational repression by the co-repressor Crc in *Pseudomonas aeruginosa*. *Nucleic Acids Res* 49: 7075–7087

Malecka EM, Woodson SA (2021) Stepwise sRNA targeting of structured bacterial mRNAs leads to abortive annealing. *Mol Cell* 81: 1988–1999

Miller JH (1972) *Experiments in molecular genetics*. Cold Spring Harbor, NY: Cold Spring Harbor Press

Milojevic T, Grishkovskaya I, Sonnleitner E, Djinovic-Carugo K, Bläsi U (2013) The *Pseudomonas aeruginosa* catabolite repression control protein Crc is devoid of RNA binding activity. *PLoS One* 8: e64609

Murshudov GN, Skubák P, Lebedev AA, Pannu NS, Steiner RA, Nicholls RA, Vagin AA (2011) REFMAC5 for the refinement of macromolecular crystal structures. *Acta Crystallogr D Biol Crystallogr* 67: 355–367

Orans J, Kovach AR, Hoff KE, Horstmann NM, Brennan RG (2020) Crystal structure of an *Escherichia coli* Hfq core (residues 2–69)-DNA complex reveals multifunctional nucleic acid binding sites. *Nucleic Acids Res* 48: 3987–3997

Pei XY, Dendooven T, Sonnleitner E, Chen S, Bläsi U, Luisi BF (2019) Architectural principles for Hfq/Crc-mediated regulation of gene expression. *Elife* 8: e43158

Pettersen EF, Goddard TD, Huang CC, Couch GS, Greenblatt DM, Meng EC, Ferrin TE (2004) UCSF Chimera - a visualization system for exploratory research and analysis. *J Comput Chem* 25: 1605–1612

Pfaffl MW (2001) A new mathematical model for relative quantification in real-time RT-PCR. *Nucleic Acids Res* 29: e45

Punjani A, Rubinstein JL, Fleet DJ, Brubaker MA (2017) cryoSPARC: algorithms for rapid unsupervised cryo-EM structure determination. *Nat Methods* 14: 290–296

Punjani A, Zhang H, Fleet DJ (2020) Non-uniform refinement: Adaptive regularization improves single particle cryo-EM reconstruction. *Nat Methods* 17: 1214–1221

Pusic P, Sonnleitner E, Bläsi U (2021) Specific and global RNA regulators in *Pseudomonas aeruginosa*. *Int J Mol Sci* 22: 8632

Pusic P, Sonnleitner E, Krennmayr B, Heitzinger DA, Wolfinger MT, Resch A, Bläsi U (2018) Harnessing metabolic regulation to increase Hfq-dependent antibiotic susceptibility in *Pseudomonas aeruginosa*. *Front Microbiol* 9: 2709

Pusic P, Tata M, Wolfinger MT, Sonnleitner E, Häussler S, Bläsi U (2016) Cross-regulation by CrcZ RNA controls anoxic biofilm formation in *Pseudomonas aeruginosa*. *Sci Rep* 6: 39621

Ramírez-Aportela E, Vilas JL, Melero R, Conesa P, Martínez M, Maluenda D, Mota J, Jiménez A, Vargas J, Marabini R *et al* (2020) Automatic local resolution-based sharpening of cryo-EM maps. *Bioinformtics* 36: 765–772

Rist M, Kertesz MA (1998) Construction of improved plasmid vectors for promoter characterization in *Pseudomonas aeruginosa* and other gram-negative bacteria. *FEMS Microbiol Lett* 169: 179–183

Rodgers ML, Woodson SA (2019) Transcription increases the cooperativity of ribonucleoprotein assembly. *Cell* 179: 1370–1381.e12

Rojo F (2010) Carbon catabolite repression in *Pseudomonas*: optimizing metabolic versatility and interactions with the environment. *FEMS Microbiol Rev* 34: 658–684

Romero M, Silistre H, Lovelock L, Wright VJ, Chan KG, Hong KW, Williams P, Camara M, Heeb S (2018) Genome-wide mapping of the RNA targets of the *Pseudomonas aeruginosa* riboregulatory protein RsmN. *Nucleic Acids Res* 46: 6823–6840

Santiago-Frangos A, Woodson SA (2018) Hfq chaperone brings speed dating to bacterial sRNA. *Wiley Interdiscip Rev RNA* 9: e1475

Schnider-Keel U, Seematter A, Maurhofer M, Blumer C, Duffy B, Gigo-Bonnefoy C, Reimmann C, Notz R, Défago G, Haas D *et al* (2000) Autoinduction of 2,4-diacetylphloroglucinol biosynthesis in the biocontrol agent *Pseudomonas fluorescens* CHAO and repression by the bacterial metabolites salicylate and pyoluteorin. *J Bacteriol* 182: 1215–1225

Schubert M, Lapouge K, Duss O, Oberstrass FC, Jelesarov I, Haas D, Allain FH (2007) Molecular basis of messenger RNA recognition by the specific bacterial repressing clamp RsmA/CsrA. *Nat Struct Mol Biol* 14: 807–813

Sonnleitner E, Abdou L, Haas D (2009) Small RNA as global regulator of carbon catabolite repression in *Pseudomonas aeruginosa*. *Proc Natl Acad Sci USA* 106: 21866–21871

Sonnleitner E, Bläsi U (2014) Regulation of Hfq by the RNA CrcZ in *Pseudomonas aeruginosa* carbon catabolite repression. *PLoS Genet* 10: e1004440

Sonnleitner E, Hagens S, Rosenau F, Wilhelm S, Habel A, Jäger KE, Bläsi U (2003) Reduced virulence of a *hfq* mutant of *Pseudomonas aeruginosa* O1. *Microb Pathog* 35: 217–228

Sonnleitner E, Pusic P, Wolfinger MT, Bläsi U (2020) Distinctive regulation of carbapenem susceptibility in *Pseudomonas aeruginosa* by Hfq. *Front Microbiol* 11: 1001

Sonnleitner E, Schuster M, Sorger-Domenigg T, Greenberg EP, Bläsi U (2006) Hfq-dependent alterations of the transcriptome profile and effects on quorum sensing in *Pseudomonas aeruginosa*. *Mol Microbiol* 59: 1542–1558

Sonnleitner E, Wulf A, Campagne S, Pei XY, Wolfinger MT, Forlani G, Prindl K, Abdou L, Resch A, Allain FH *et al* (2018) Interplay between the catabolite repression control protein Crc, Hfq and RNA in Hfq-dependent translational regulation in *Pseudomonas aeruginosa*. *Nucleic Acids Res* 46: 1470–1485

Tegunov D, Cramer P (2019) Real-time cryo-electron microscopy data preprocessing with Warp. *Nat Methods* 16: 1146–1152

Trouillon J, Han K, Attrée I, Lory S (2022) The core and accessory Hfq interactomes across *Pseudomonas aeruginosa* lineages. *Nat Commun* 13: 1258

Valentini M, Garcia-Maurino SM, Perez-Martinez I, Santero E, Canosa I, Lapouge K (2014) Hierarchical management of carbon sources is regulated similarly by the CbrA/B systems in *Pseudomonas aeruginosa* and *Pseudomonas putida*. *Microbiology* 160: 2243–2252

 © 2022 The Authors

Williams C, Headd J, Moriarty N, Prisant M, Videau L, Deis L, Verma V, Keedy D, Hintze B, Chen V *et al* (2017) MolProbity: More and better reference data for improved all-atom structure validation. *Protein Sci* 27: 293–315

Winsor GL, Griffiths EJ, Lo R, Dhillon BK, Shay JA, Brinkman FSL (2016) Enhanced annotations and features for comparing thousands of *Pseudomonas* genomes in the *Pseudomonas genome database. Nucleic Acids Res* 44: 646–653

Yang N, Ding S, Chen F, Zhang X, Xia Y, Di H, Cao Q, Deng X, Wu M, Wong C *et al* (2015) The Crc protein participates in down-regulation of the *lon* gene to promote rhamnolipid production and quorum sensing in *Pseudomonas aeruginosa*. *Mol Microbiol* 96: 526–547

Yu AM, Gasper PM, Cheng L, Lai LB, Kaur S, Gopalan V, Chen AA, Lucks JB (2021) Computational reconstructing cotranscriptional RNA folding from experimental data reveals rearrangement of non-native folding intermediates. *Mol Cell* 81: 870–883

Zhang A, Schu DJ, Tjaden BC, Storz G, Gottesman S (2013) Mutations in interaction surfaces differentially impact *E. coli* Hfq association with small RNAs and their mRNA targets. *J Mol Biol* 425: 3678–3697

Zhang YF, Han K, Chandler CE, Tjaden B, Ernst RK, Lory S (2017) Probing the sRNA regulatory landscape of *Pseudomonas aeruginosa*: post-transcriptional control of determinants of pathogenicity and antibiotic susceptibility. *Mol Microbiol* 106: 919–937

Zuker M (2003) Mfold web server for nucleic acid folding and hybridization prediction. *Nucleic Acids Res* 31: 3406–3415

License: This is an open access article under the terms of the Creative Commons Attribution License, which permits use, distribution and reproduction in any medium, provided the original work is properly cited.

Expanded View Figures

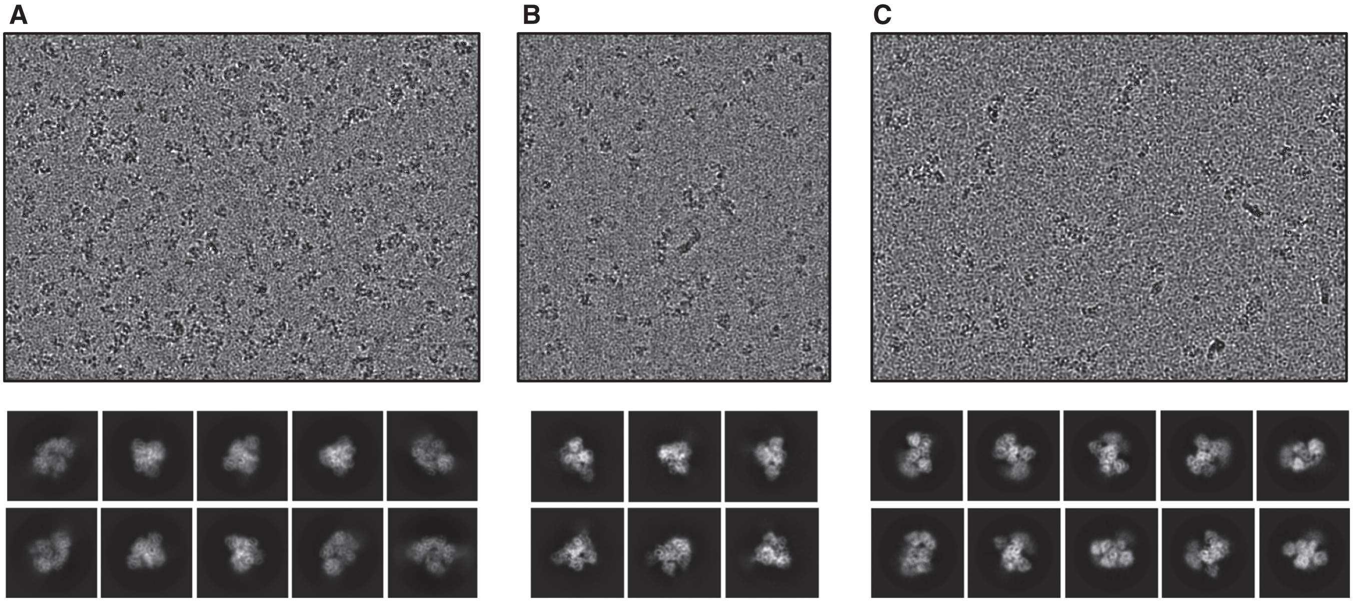

Figure EV1. **Raw images and 2D class averages of the Hfq–Crc translation repression complexes formed on *amiE*$_{105}$ (A), *estA*$_{118}$ (B), and *rbsB*$_{110}$ (C).**

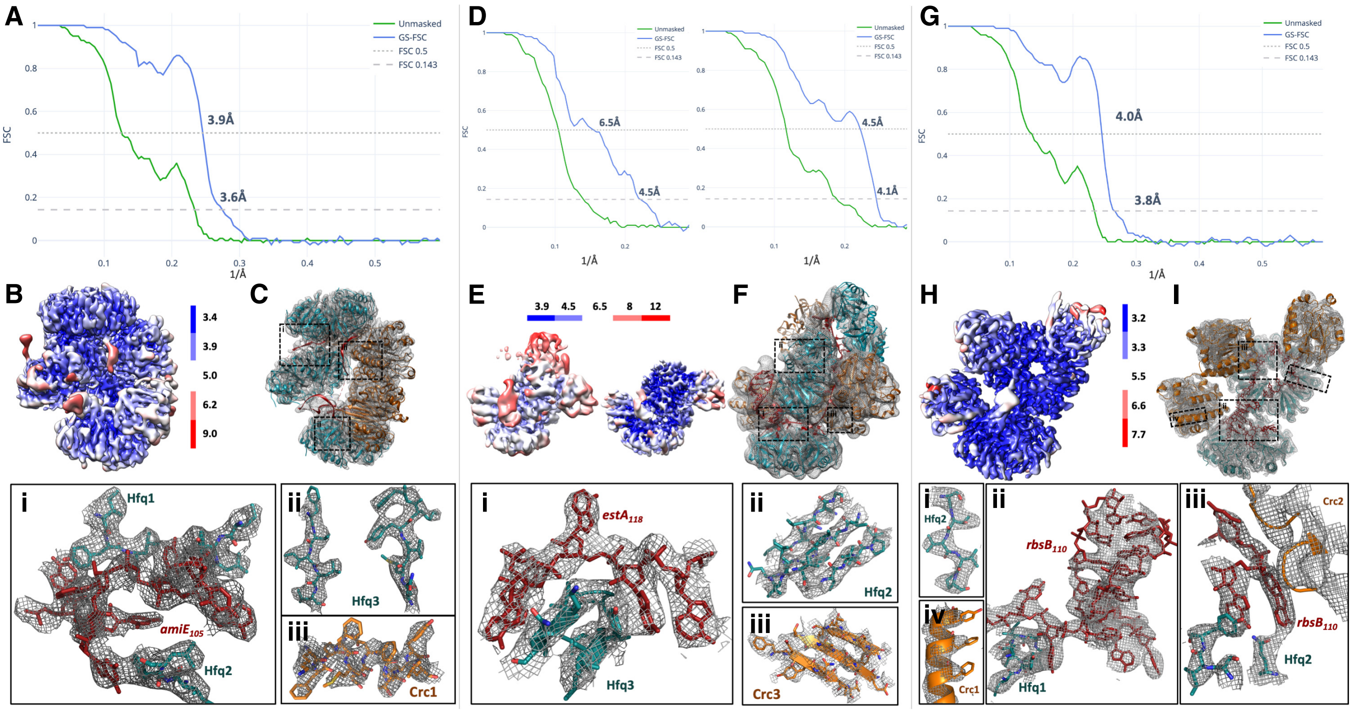

Figure EV2.

 © 2022 The Authors

Figure EV2. Global and local resolution analyses of the Hfq–Crc translation repression complexes formed on *amiE*$_{105}$, *estA*$_{118}$, and *rbsB*$_{110}$.

A–I (A), (D), and (G) show global FSC curves (gold standard) for the *amiE*$_{105}$, *estA*$_{118}$, and *rbsB*$_{110}$ complexes, respectively. The left FSC curve in panel (D) corresponds to the global, consensus refinement; the right FSC curve corresponds to the focused refinement for the Hfq–estA118–Crc reconstruction. (B), (E), and (H) display local resolution estimates as measured by cryoSPARC at FSC 0.5. (C), (F), and (I) show the respective structures, colored as before, docked into the experimental cryo-EM maps, with the insets showing close-up of selected areas for each.

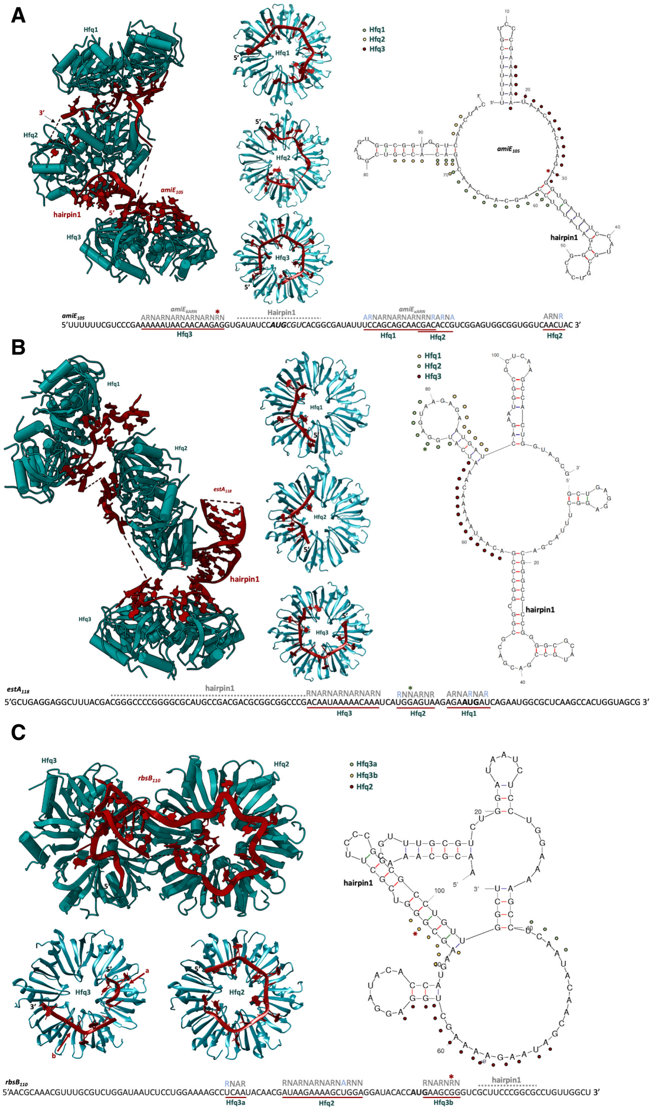

Figure EV3. Recognition and presentation of *amiE*$_{105}$, *estA*$_{119}$, and *rbsB*$_{110}$ by Hfq.

A *amiE*$_{105}$ is presented by three Hfq hexamers and adopts complete or partial ARN motif engagement on each distal side. The proximal side of Hfq2 coordinates an amiE105 hairpin-loop structure (hairpin1). A second hairpin-loop forms at the 3′-end of amiE105 on the Hfq2 distal side (hairpin2, not shown due to limited resolution). Right: annotated secondary structure prediction of *amiE*$_{105}$ (mfold; Zuker, 2003). Colored dots indicate which Hfq distal side presents the ARN-rich motif in the Hfq–*amiE*$_{105}$–Crc model. An annotated sequence is depicted at the bottom of the panel. Sequences that were mapped in the cryo-EM reconstruction are underlined in red and the Hfq distal sides they bind to are labeled in green. Occupied A-, R-, or N-sites are annotated in gray above each modeled sequence. * refers to an A-site "skipping"-violation, where the A-site on the Hfq distal site is not occupied by a base, i.e., skipped. Light blue letters refer to "mismatch"-violations of the ARN rule, where a pyrimidine base occupies an A-site or R-site pocket on the Hfq distal face. The ranges for hairpin1 and hairpin2 are arbitrary due to limited local resolution in the corresponding map regions.

B *estA*$_{118}$ is presented by three Hfq hexamers and adopts partial ARN motif engagement on each Hfq distal side. The proximal side of Hfq2 coordinates an estA118 hairpin-loop structure (hairpin 1). Right: annotated secondary structure prediction of *estA*$_{118}$ (mfold; Zuker, 2003). Colored dots indicate which Hfq distal side presents the ARN-rich motif in the Hfq–Crc–*estA*$_{118}$ model. An annotated sequence is depicted at the bottom of the panel. Sequences that were mapped in the cryo-EM reconstruction are underlined in red and the Hfq distal sides they bind to are labeled in green. Occupied A-, R-, or N-sites are annotated in gray above each modeled sequence. The * refers to an A-site "skipping"-violation, where the A-site on the Hfq distal site is not occupied by a base, i.e., skipped. Light blue letters refer to "mismatch"-violations of the ARN rule, where a pyrimidine base occupies an A-site or R-site pocket on the Hfq distal face.

C *rbsB*$_{110}$ is presented by three Hfq hexamers (only the two that were well resolved in the cryo-EM maps are shown) and adopts partial ARN motif engagement on each Hfq distal side. The proximal side of Hfq2 coordinates a *rbsB*$_{110}$ hairpin-loop structure (hairpin1, in the back of the Hfq2 hexamer, not annotated in the figure). Right: annotated secondary structure prediction of *rbsB*$_{110}$ (mfold; Zuker, 2003). Colored dots indicate which Hfq distal side presents the ARN-rich motif in the Hfq–*rbsB*$_{110}$–Crc model. An annotated sequence is depicted at the bottom of the panel. Sequences that were mapped in the cryo-EM reconstruction are underlined in red and the Hfq distal sides they bind to are labeled in green. Occupied A-, R-, or N-sites are annotated in gray above each modeled sequence. * refers to an A-site "skipping"-violation, where the A-site on the Hfq distal site is not occupied by a base, i.e., skipped. Light blue letters refer to "mismatch"-violations of the ARN rule, where a pyrimidine base occupies an A-site or R-site pocket on the Hfq distal face. The range for hairpin1 is arbitrary due to limited local resolution in the corresponding map region.

© 2022 The Authors
