# Supplementary material for: Translational regulation by Hfq–Crc assemblies emerges from polymorphic ribonucleoprotein folding
Source: EMBO J. 2022 Dec 12;42(3):e111129. doi: 10.15252/embj.2022111129 (PMC9890229; doi:10.15252/embj.2022111129)
Supplement: Supplementary file 4 [file EMBJ-42-e111129-s004.pdf]

THE EMBO JOURNAL

Translational regulation by Hfq-Crc assemblies emerges from polymorphic ribonucleoprotein folding

Tom Dendooven, Elisabeth Sonnleitner, Udo Bläsi and Ben Luisi
DOI: 10.15252/embj.2022111129

Corresponding author(s): Ben Luisi (bfl20@cam.ac.uk)

Review Timeline:

Submission Date:	10th Mar 22
Editorial Decision:	2nd May 22
Revision Received:	8th Sep 22
Editorial Decision:	19th Oct 22
Revision Received:	25th Oct 22
Accepted:	11th Nov 22

Editor: Stefanie Boehm

Transaction Report:

(Note: With the exception of the correction of typographical or spelling errors that could be a source of ambiguity, letters and reports are not edited. Depending on transfer agreements, referee reports obtained elsewhere may or may not be included in this compilation. Referee reports are anonymous unless the Referee chooses to sign their reports.)

Thank you for submitting your manuscript EMBOJ-2022-111129 to The EMBO Journal. We have now received three referee reports on your study, which are included below for your information. In light of these comments, we would like to invite you to prepare and submit a revised manuscript.

As you will see, the reviewers are overall positive and appreciate the findings and their interest to the field. However, they do raise a number of points which should be addressed in the revised version of the manuscript. In particular, the proposed effect of Hfq-Hfq polymerization on translational repression (ref#1- point 5) should be further assessed and referee #2' major concern regarding the stoichiometry of Hfq and Crc resolved (also ref#1- point 1, 6). Please also carefully consider all other referee comments and revise the manuscript and figures as needed, as well as providing a detailed response to each comment.

Please note that it is our policy to allow only a single round of major revision. Acceptance depends on a positive outcome of a second round of review and therefore on the completeness of your responses included in the next, final version of the manuscript. I encourage you to review the referees' comments and contact me to discuss any questions or a preliminary revision plan in case there are any uncertainties regarding specific points or the revision in general.

Referee #1:

Hfq, known to be an important RNA chaperone, particularly in many gram negative bacteria, plays a unique role in modulating carbon catabolite repression in Pseudomonas in collaboration with a second critical protein, Crc. Hfq and Crc together are known to form repressive complexes on a variety of mRNAs for secondary carbon sources; these mRNAs are only released to be translated when the repression is relieved by a competing sRNA, CrcZ. Previously, this group (Pei et al, 2019) presented the cryoEM structure of Hfq and Crc on one target mRNA, amiE, and demonstrated the importance of repeated ARN motifs in binding Hfq, finding a number of different complexes with different stoichiometry of Hfq, Crc and mRNA. Here the authors extend that previous work, both using a longer version of the amiE mRNA (with more ARNs) and comparing complexes on two additional mRNAs with somewhat different arrangements of ARN sequences. The results are of significant interest in reflecting the flexibility of the interactions, and to some extent, that lesson is stronger than evidence for specific complexes being formed and necessary for translational repression in vivo. A few problems or issues that could be further discussed are noted. In particular, while some of the predicted interactions are tested with appropriate mutants, the interpretations of the results with those mutants are not always convincing. Second, given the different degrees to which Hfq and Hfq with the help of Crc repress the three tested targets, and the somewhat different geometrys of repression observed, it would have been useful to understand if this relates at all to the stability or extent of repression - is this chance or a biologically relevant variation in how these complexes can form?

1. Fig. 3A: amiE sequences and higher order complexes: In the previous work, a 2:2:2 complex was the primary version seen, and suggested to be an "all or nothing" complex, with the crc dimer sandwiched between the hfq/amiE sequences, presenting the same faces to the two Hfqs. In 3A, it would seem that the Crc/Crc dimerization interface is not clearly present. Is that correct? Is this clearly due to the longer amiE sequence or other differences in assembling and analyzing complexes? If that interface is not present, does it form in the higher order structures? Do any of the results here affect the interpretation of mutants in that dimerization interface from Pei et al?
2. Line 245-246, Fig. 4B; Why is the "core" unit described as two amiE105 copies with a Hfq-Crc-Crc? Or is that actually two separate versions of Hfq-amiE-Crc-Crc, as it seems to be in 4B?
3. Fig. 4H: Text (lines 250-253) and figure are a bit unclear on exactly what mutants were tested. Please clarify. R140E shows lack of repression for amiE, not listed in test (R140A is mentioned). Was R140A R141A tested in an otherwise WT background (listed as R140/1A? not shown on these tests), or only the quadruple mutant (R140/1A R138/K39A)? Evidence that these proteins are stably produced and folded?
4. Expanding on the issue of Crc/Crc dimers, Fig. 4E and 4G show three different Crc interfaces. The text and the 4E suggests that the amiE complex has two of these, while none are present for estA, and 3 is present for rbsB. Is that correct? As far as I can tell, none of these are athe same as the two discussed in Pei et al, although R230 was shown in one of these, and R230E did not have a phenotype for amiE regulation in that paper. Here, Fir. 4H shows that E193R is defective but that E193R R230E is not. I didn't see E193 interacting with R230 in any of the three interfaces. Why are these then leading to suppression? The authors argue that the striking phenotype of these mutants for estA is due to changes in Hfq interactions (lines 300-302). Are those residues unable to have the same negative and positive effects with Hfq for the other targets? Similarly for R90E Y91F: the effects are as strong for complexes without the interface as those with it, and then some other effect is invoked. sensitivity to R90E Y91F? Without further mutational analysis (effects of Hfq mutations, possibly, or other mutations in these regions), it is not possible to conclude that these mutations reflect the importance of the Crc interface.
5. The authors consider the RNA-based Hfq-Hfq polymerization as an important component of efficient translational repression. Some of this could use further work to be fully convincing.

a. Lines 317-320: The loss of the sites downstream from the AUG would seem to have a reasonably modest effect (Fig. 2D);

does it change the effectiveness of induction by CrcZ or degree of dependence on Crc? Is the expectation that the complex seen in Pei et al (with a short RNA region) would be what would form in this case? In the data in Fig. 4H, loss of Hfq has a much stronger effect than loss of Crc; that is not so clearly the case for amiE (although it is a bit hard to tell with the log scale used here). I see this point is returned to in the discussion, but there, the significant repression is emphasized, while in this earlier part, it seems to suggest that these shared sites are important. Possibly testing a fusion with the sites mutated rather than deleted would be informative.

b. Fig. 5J, estA repression: Is it clear that Hfq binds/represses estA at all in the absence of the Hfq3 binding site? This could be tested by gel shift, and/or in vivo, comparing Hfq+ to loss of Hfq for this mutant. Crc effect is mostly lost, but is that because Hfq binding is lost entirely?

c. Fig. 5I, rbsB presentation to Hfq rim site. Do Hfq rim mutants affect efficiency of rbsB represssion? Is that specific to rbsB?

6. A few points that might be expanded on in the discussion: Do the authors think that these different complexes provide different degrees of stability of repression? Is there a hierarchy that helps CCR function efficiently based on the nature of these complexes? If repression leads to degradation, presumably these complexes are not reversible. Is that known to be the case? Does induction by CrcZ simply prevent them from forming? The authors suggest that if transcripts are degraded once repressed, that would allow recycling of Hfq and Crc; they also mention the possibility of transcription termination occurring if repression is co-transcriptional. Is it clear whether ribonucleases would be able to access the RNA within this complex? Might less 3' mRNA reflect exonucleases unable to degrade into the complex?

7. Line 198: Shouldn't the stoichiometry here be 1 Hfq:1 amiE: 2 Crc, as in Fig. 3A (says 2:1:1 in text)?

Referee #2:

In the present manuscript, Dendooven et al studied the molecular mechanisms underlying the interaction of Crc, Hfq, and several RNA binding partners. Previous work has shown that Crc plays an important role in CCR (carbon catabolite repression) of Pseudomonas aeruginosa and that regulation by Crc occurs at the post-transcriptional level through a mechanism involving the global RNA-binding protein, Hfq. In this work, the authors employed cryo-EM to investigate the structure of Crc in combination with Hfq and the translation initiation regions of three selected mRNA binding partners, i.e. amiE, estA, and rbsB. The authors solved the structures of all of these complexes and discovered structural polymorphism among the different assemblies, which relied on specific sequence motifs in the mRNAs. Interestingly, the obtained quaternary architectures involved several copies of Crc and Hfq forming higher order repressive complexes. Importantly, the information obtained from the cryo-EM structures is supported by extensive genetic and biochemical data. Taken together, I think this is a high quality manuscript and I have only few comments, which I listed below.

Major point of criticism:

- I have only one major comment, which refers to the stoichiometry of the reported complexes and how this would translate in the availability of Hfq and Crc in the cell. Specifically, Hfq has been reported to be limiting in the cell constantly cycling between different RNP complexes (e.g. Fender et al, Genes Dev., PMID: 21123649) and I wondered how these data can be corroborated with the findings in this manuscript. This question is briefly addressed in the discussion section (lines 428-434), however, it is not clear to me how ribonucleases would initiate turn-over of the complex (would they have access the relevant RNA segments in the first place?).

Minor points of criticism:

- Line 79: suggest to change to "well-studied"
- Fig. 2C: italicize "lacZ"
- Lines 247: I thought this part was a little confusing: if Crc interacts with the phosphate backbone, how is specificity achieved?
- Line 294: italicize "crc"

Referee #3:

In 2021 the authors had already shown that "Stabilization of Hfq mediated translational repression by the co-repressor Crc in Pseudomonas aeruginosa" (2021, NAR 49, 7075-7087). They demonstrated that in this organism Hfq can collaborate with Crc protein to down-regulate translation of mRNAs that become targets for the degradative machinery. The model proposed in the last figure of this manuscript already proposed that Hfq and Crc could form higher order assemblies.

In this current paper the findings provide structural confirmation of these assemblies in different quaternary structures, and showed that the multi-step assembly process is highly cooperative and probably competes with the ribosomes repressing translation. The structural polymorphism enables selective translational repression of many different target RNAs. It is apparent that completion of higher order assembly enhances translational repression, and that RNA-mediated Hfq-Hfq oligomerization drives this process. The Hfq/Crq assembly is tuned temporally and the resulting translation repression complexes are transient in nature, subject to kinetic competition.

Most of the conclusions are well supported (with exception of the results of the deletion of an hairpin structure). However, the introduction and the discussion only focus on Pseudomonas aeruginosa and ignore many related publications on the roles of Hfq and interaction with other RNAs /proteins. This has to be substantially improved. Below I indicate several publication which should be referred in the introduction and put in context when discussing the roles of Hfq and translation.

Relevant references to be cited in this manuscript:
Trouillon J, Han K, Attrée I, Lory S. The core and accessory Hfq interactomes across Pseudomonas aeruginosa lineages. Nat Commun. 2022 Mar 10;13(1):1258.

Dendooven T, Sinha D, Roeselová A, Cameron TA, De Lay NR, Luisi BF, Bandyra KJ. A cooperative PNPase-Hfq-RNA carrier complex facilitates bacterial riboregulation.
Mol Cell. 2021 Jul 15;81(14):2901-2913.e5.

Bharwad, K. & Rajkumar, S. Rewiring the functional complexity between Crc, Hfq and sRNAs to regulate carbon catabolite repression in Pseudomonas. World J. Microbiol. Biotechnol. 35, 140 (2019).

dos Santos, R. F., Arraiano, C. M. & Andrade, J. M. New molecular interactions broaden the functions of the RNA chaperone Hfq. Curr. Genet. 65, 1313-1319 (2019).

Andrade, J. M., dos Santos, R. F., Chelysheva, I., Ignatova, Z. & Arraiano, C. M. The RNA-binding protein Hfq is important for ribosome biogenesis and affects translation fidelity. EMBO J. 37, e97631 (2018).

Chen J, Gottesman S. Hfq links translation repression to stress-induced mutagenesis in E. coli. Genes Dev. 2017 Jul 1;31(13):1382-1395.

When it is mentioned that Hfq has different interaction surfaces it has to be mentioned the first article which called the attention to this fact:
Zhang A, Schu DJ, Tjaden BC, Storz G, Gottesman S. Mutations in interaction surfaces differentially impact E. coli Hfq association with small RNAs and their mRNA targets. J Mol Biol. 2013 Oct 9;425(19):3678-97.

In this manuscript it is not explained the puzzling effect of increasing translation repression when it is deleted a stem loop before the start codon. Please try to give a better explanation taking into account related papers, and refer to them in your discussion:
Ishikawa H, Otaka H, Maki K, Morita T, Aiba H. The functional Hfq-binding module of bacterial sRNAs consists of a double or single hairpin preceded by a U-rich sequence and followed by a 3' poly(U) tail. RNA. 2012 May;18(5):1062-74.

Hopkins, J. F., Panja, S., McNeil, S. A. N. & Woodson, S. A. Effect of salt and RNA structure on annealing and strand displacement by Hfq. Nucleic Acids Res. 37, 6205-6213 (2009).

These were my major concerns and below I indicate some minor concerns during the reading of the manuscript.

Minor concerns:

1- In the beginning of the introduction (page 3) it refers that only two post-transcriptional systems control gene expression in P. aeruginosa. The sentences have to be rephrased since other important mechanisms are also quite relevant in post-transcriptonal control, such as the action of ribonucleases and the relevance of RNA degradation.

2-Line 66 you have to mention that Hfq has also been shown to be in complex with many other proteins (see above some relevant references).

3- Line 99- Refer who has discovered the three binding surfaces in Hfq- namely the paper above "Zhang et al 2013".

4-Line 360- The role of RNA stability/degradation has to be better stressed throughout the text.
Namely it has been considered that respective mRNA levels = relative translational efficiency to account for intrinsic changes in RNA stability.

5- Try to explain better why to remove an hairpin structure has significantly stronger translation repression.

6- Fig5- It is said that mutations are indicated in green, but that is not clear.

Referee #1:

Hfq, known to be an important RNA chaperone, particularly in many gram negative bacteria, plays a unique role in modulating carbon catabolite repression in Pseudomonas in collaboration with a second critical protein, Crc. Hfq and Crc together are known to form repressive complexes on a variety of mRNAs for secondary carbon sources; these mRNAs are only released to be translated when the repression is relieved by a competing sRNA, CrcZ. Previously, this group (Pei et al, 2019) presented the cryoEM structure of Hfq and Crc on one target mRNA, amiE, and demonstrated the importance of repeated ARN motifs in binding Hfq, finding a number of different complexes with different stoichiometry of Hfq, Crc and mRNA. Here the authors extend that previous work, both using a longer version of the amiE mRNA (with more ARNs) and comparing complexes on two additional mRNAs with somewhat different arrangements of ARN sequences. The results are of significant interest in reflecting the flexibility of the interactions, and to some extent, that lesson is stronger than evidence for specific complexes being formed and necessary for translational repression in vivo. A few problems or issues that could be further discussed are noted. In particular, while some of the predicted interactions are tested with appropriate mutants, the interpretations of the results with those mutants are not always convincing. Second, given the different degrees to which Hfq and Hfq with the help of Crc repress the three tested targets, and the somewhat different geometrys of repression observed, it would have been useful to understand if this relates at all to the stability or extent of repression - is this chance or a biologically relevant variation in how these complexes can form?

Our reply: We thank the reviewer for these comments, as well as those that follow below. Regarding the relationship between geometry, stability and extent of repression, our conjecture is that the quaternary states represent a biologically relevant variation that impacts on the kinetics and stability of the complexes. Our recent work with Sarah Woodson and colleagues demonstrates the impact of Crc on the kinetics of higher order assembly, which provides a first glimpse on how complex stability and readout is linked to assembly and quaternary structure (Malecka, et al. (2021) Stabilization of Hfq-mediated translational repression by the co-repressor Crc in *Pseudomonas aeruginosa. Nucleic Acids Res.* **49**, 7075-7087). As regulation is a non-equilibrium process, the variation in assembly is likely to have biological meaning in the graded readout of translational repression.

1. Fig. 3A: amiE sequences and higher order complexes: In the previous work, a 2:2:2 complex was the primary version seen, and suggested to be an "all or nothing" complex, with the crc dimer sandwiched between the hfq/amiE sequences, presenting the same faces to the two Hfqs. In 3A, it would seem that the Crc/Crc dimerization interface is not clearly present. Is that correct? Is this clearly due to the longer amiE sequence or other differences in assembling and analyzing complexes? If that interface is not present, does it form in the higher

order structures? Do any of the results here affect the interpretation of mutants in that dimerization interface from Pei et al?

Our reply: We agree with the reviewer that the Crc/Crc dimerization interface seen in the previous 2:2:2 complex with the short amiE RNA (18-mer) is not present in the new complex for the longer RNA. The Crc:Crc interface seen earlier appears to be disrupted with the longer *amiE* RNA, which permits an Hfq hexamer (Hfq2) to fit into the space that would have otherwise been filled with the dimerising Crc/Crc pair. Because those surfaces form other interactions, the mutants in the dimerization interface described in Pei *et al*. still impact on other interactions made by Crc in the complexes studied here. Crc E142 for example does not participate in Crc dimer interface formation in the complexes formed on the longer *amiE*, but now forms contacts with Hfq2, which explains its relevance in vivo observed in the Pei *et al.* paper. However, the Crc dimer interface observed in the 2:4:2 complex (Figure 4A in the Pei *et al.* paper, Crc3-4, E193) overlaps with dimer interface 2 in the complex formed on the longer *amiE* (Figure 4G in this manuscript, interface 2). We show that a E193R mutation abrogates repression in vivo. Furthermore, the core interactions of Crc/Hfq seen in the 1:1:2 complex in Figure 3A and Figure 4C/D remain the same as in the earlier reported 2:2:2, 2:4:2 and 2:3:2 complexes with the short *amiE* 18-mer, and that appears to be the fundamental unit upon which higher order assemblies form.

We thank the reviewer for raising this point, and we have changed the text to explain the complexity.

2. Line 245-246, Fig. 4B; Why is the "core" unit described as two amiE105 copies with a Hfq-Crc-Crc? Or is that actually two separate versions of Hfq-amiE-Crc-Crc, as it seems to be in 4B?

Our reply: We propose that the core unit is the Hfq-Crc-Crc shown in Figure 3A on the left panel and described in response to point 1 above. It is not two copies of *amiE105*, but two copies of the core unit the form on one *amiE105* RNA, one on each ARN-motif. We apologise that this was not clear from the wording, and we have revised the figure legend and text to explain this better.

3. Fig. 4H: Text (lines 250-253) and figure are a bit unclear on exactly what mutants were tested. Please clarify. R140E shows lack of repression for amiE, not listed in test (R140A is mentioned). Was R140A R141A tested in an otherwise WT background (listed as R140/1A? not shown on these tests), or only the quadruple mutant (R140/1A R138/K39A)? Evidence that these proteins are stably produced and folded?

Our reply: The mutations tested (now mentioned in lines 271-273 are R140E, and the quadruple mutant R138A/K139A/R140A/R141A. We have changed the text here to make this clearer. We have showed in Pei *et al*. that these proteins are stably produced and folded.

4. Expanding on the issue of Crc/Crc dimers, Fig. 4E and 4G show three different Crc interfaces. The text and the 4E suggests that the amiE complex has two of these, while none are present for estA, and 3 is present for rbsB. Is that correct? As far as I can tell, none of these are athe same as the two discussed in Pei et al, although R230 was shown in one of these, and R230E did not have a phenotype for amiE regulation in that paper. Here, Fir. 4H shows that E193R is defective but that E193R R230E is not. I didn't see E193 interacting with R230 in any of the three interfaces. Why are these then leading to suppression?
The authors argue that the striking phenotype of these mutants for estA is due to changes in Hfq interactions (lines 300-302). Are those residues unable to have the same negative and positive effects with Hfq for the other targets? Similarly for R90E Y91F: the effects are as strong for complexes without the interface as those with it, and then some other effect is invoked. sensitivity to R90E Y91F? Without further mutational analysis (effects of Hfq mutations, possibly, or other mutations in these regions), it is not possible to conclude that these mutations reflect the importance of the Crc interface.

Our reply: We thank the reviewer for spotting these potential irregularities in the data presentation. Replying to the E193/R230 points: Interfaces 2 and 3 in Figure 4E partially overlap with the Crc3/4 interface in the Pei *et al.* paper, as some of the same amino acids, such as E193, participate the interface. For interface 2 in the *amiE* complex we don't see convincing sidechain density for R230 to suggest it interacts with E193 (which does not mean it doesn't), but in interface 3 in the *rbsB* complex R230 forms contacts with E193 in Crc1. We are not showing this contact in Figure 4G because it is not directly part of an inter-Crc interface (but rather an intra-Crc interface). We postulate that in the *rbsB* complex the double mutation E193R R230E is compensatory for E193R and will interact with E61 and R90 respectively, resulting in translation repression. In the *amiE* and *rbsB* assemblies neither E193 nor R230 form contacts with any Hfq hexamers, meaning that the observed phenotype for the E193R and E193R/R230E mutants can be attributed solely to the disruption of Crc-Crc interface 2 and 3 in the *amiE* and *rbsB* complexes, respectively.

In regard to the points about R90/Y9: In the *rbsB* assemblies neither E90 nor Y91 form contacts with Hfq protomers, meaning that the observed phenotype for the R90E/Y91F double mutant can be attributed solely to the disruption of Crc-Crc interface 3 in the *rbsB* complex.

In summary, we tried to identify mutations that would selectively impact on only one of the three quaternary structures, but in all cases that we explored the same residues are used in different ways and mutations therefore impact on all assemblies. While we exhausted Crc mutations, we did not try Hfq mutants, as it has been observed in our lab by other groups that such mutations cause pleiotropic riboregulatory effects in the cell, making it hard to attribute phenotypes to specific molecular pathways.

We agree that the results of these *in vivo* studies were not described adequately in the text and have changed the wording to clarify things further. We hope that the explanation above and changes to the text show the importance of the Crc interfaces for adequate formation the respective complexes *in vivo*.

5. The authors consider the RNA-based Hfq-Hfq polymerization as an important component of efficient translational repression. Some of this could use further work to be fully convincing.
a. Lines 317-320: The loss of the sites downstream from the AUG would seem to have a reasonably modest effect (Fig. 2D); does it change the effectiveness of induction by CrcZ or degree of dependence on Crc? Is the expectation that the complex seen in Pei et al (with a short RNA region) would be what would form in this case? In the data in Fig. 4H, loss of Hfq has a much stronger effect than loss of Crc; that is not so clearly the case for amiE (although it is a bit hard to tell with the log scale used here). I see this point is returned to in the discussion, but there, the significant repression is emphasized, while in this earlier part, it seems to suggest that these shared sites are important. Possibly testing a fusion with the sites mutated rather than deleted would be informative.

Our reply: We explored the downstream site of *amiE* by mutating the A-site of the secondary $amiE_{xARN}$ motif that is bound by the secondary Hfq (see figure below). We did not explore the effectiveness of induction by CrcZ or degree of dependence on Crc, but all the experiments were done in the presence of succinate, *i.e.* under conditions when Hfq/Crc- mediated repression is in effect. Both of these proposed experiments are good suggestions and have been planned for follow-up studies. Trimming down *amiE* to remove the secondary ARN motif, as we did in this study could lead to formation of two complexes *in vivo*. One is indeed the complex observed in Pei *et al.,* although 2 mRNA species would have to come together in the cell for this. A second complex could be the Hfq-Crc-Crc core as shown in Figure 3A.

Contrary to our predictions, the translational reporter system records stronger repression for all three sets of mutations tested (results shown below). Although we did not expect strong phenotypes for A-to-C and A-to-T mutations in the $amiE_{\mathrm{xARN}}$ region, a decrease in repression was expected for A-to-G mutations, since A-site pocket on the Hfq distal side cannot accommodate Guanine bases. We note that the mutations increase the strength of predicted secondary structure in the transcript within the proposed secondary Hfq binding site (right top panel in figure below), in particular for A-to-G. We wonder if the fold of the RNA confounds the interpretation of the results. The translational efficiency (ratio of transcripts to beta-galactosidase activity) is the same for all transcripts within error, suggesting that the RNA changes do not interfere with the ability of the transcripts to be translated. The potential confounding impact of RNA structure is also related to the point raised by reviewer 3 regarding the increase translational repression seen for the *estA* construct, in which the hairpin is removed.

In light of these results, we have added text to the discussion section regarding the role of secondary structure in the RNA and its impact on Hfq binding (Lines 438-445).

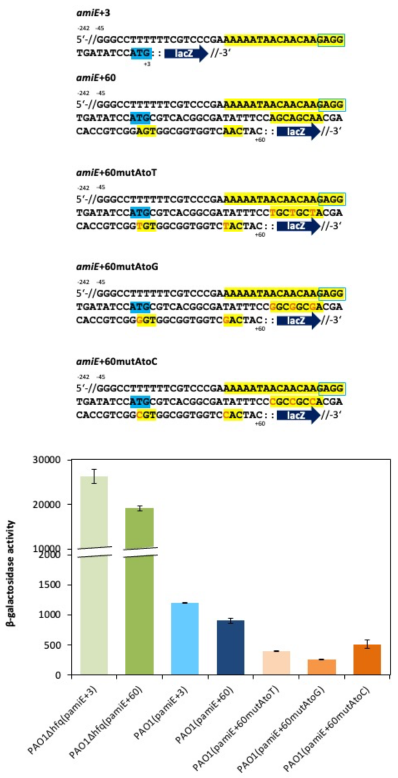

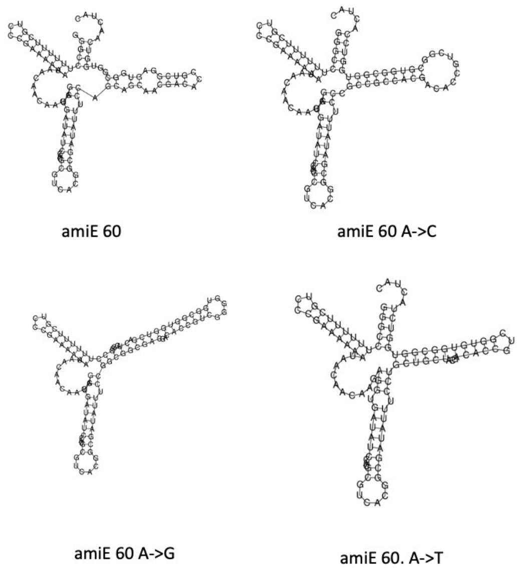

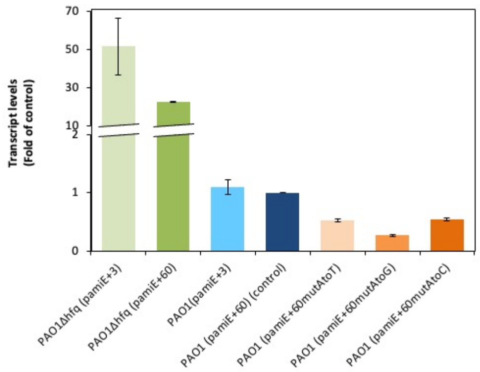

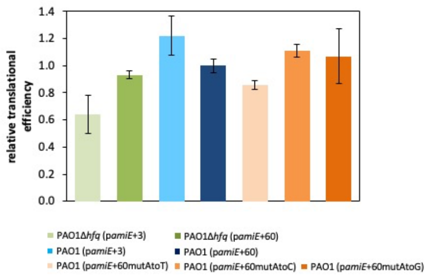

b. Fig. 5J, estA repression: Is it clear that Hfq binds/represses estA at all in the absence of the Hfq3 binding site? This could be tested by gel shift, and/or in vivo, comparing Hfq+ to loss of Hfq for this mutant. Crc effect is mostly lost, but is that because Hfq binding is lost entirely?

Our reply: When compared with strain PAO1*Δcrc*(pME4510crc$_{Flag}$, pestA+18:::lacZ) (*estA* wt sequence), some translational repression is retained after elimination of the Hfq3 (Hfq3mut) binding element in strain PAO1*Δcrc*(pME4510crc$_{Flag}$, pestA+13ΔCA:::*lacZ*). Thus, although the Hfq3 binding site is required for repression, it is unlikely that Hfq binding is completely lost when it is mutated, i.e. via the Hfq1/2 RNA fragments. In addition, hairpin1 is likely to bind the Hfq2 proximal side still in the absence of the Hfq3 binding motif, as shown in Figure 5E.

c. Fig. 5I, rbsB presentation to Hfq rim site. Do Hfq rim mutants affect efficiency of rbsB represssion? Is that specific to rbsB?

Our reply: This is a good point. We have not tested Hfq rim mutants *in vivo*, but are planning to do this in future studies. The mutation is likely to have many off target effects that may confound the analysis and will require extensive validation.

6. A few points that might be expanded on in the discussion: Do the authors think that these different complexes provide different degrees of stability of repression? Is there a hierarchy that helps CCR function efficiently based on the nature of these complexes? If repression leads to degradation, presumably these complexes are not reversible. Is that known to be the case? Does induction by CrcZ simply prevent them from forming? The authors suggest that if transcripts are degraded once repressed, that would allow recycling of Hfq and Crc; they also mention the possibility of transcription termination occurring if repression is co-transcriptional. Is it clear whether ribonucleases would be able to access the RNA within this complex? Might less 3' mRNA reflect exonucleases unable to degrade into the complex?

Our reply: We thank the reviewer for these insightful questions. Regarding the different degrees of stability of repression, we note that many genes during CRC are repressed to different degrees. In *Pseudomonas putida*, changes in metabolic fluxes are observed in the absence of Crc, indicating a hierarchy of repression. Regarding the reversibility, the complexes may be stable, but it is difficult to test 'assembly turnover' *in vivo and* we did not explore this. Our recent molecular dynamics study with Jiri Sponer (Krepl *et al.,* 2021*)* on RNA cycling on the Hfq distal side suggests that such cycling is significantly attenuated when Crc binds to the RNA presented by Hfq. The latter indicates that the assemblies might not be reversible indeed. We note that the complexes are at the 5' end, and could protect from endonucleases, but 3'-5' exoribonucleases could attack the transcript. As a general principle, following translational repression the RNA will be attacked by

nucleases. We have added to the discussion section the following additional references to relevant papers (Corona et al., 2018; Rojo et al.,2010; Malecka et al., 2021; Iost and Dreyfus, 1995).

7. Line 198: Shouldn't the stoichiometry here be 1 Hfq:1 amiE: 2 Crc, as in Fig. 3A (says 2:1:1 in text)?

Our reply: Indeed, the stoichiometry should be 1:1:2. We have corrected this in the modified text.

Referee #2:

In the present manuscript, Dendooven et al studied the molecular mechanisms underlying the interaction of Crc, Hfq, and several RNA binding partners. Previous work has shown that Crc plays an important role in CCR (carbon catabolite repression) of Pseudomonas aeruginosa and that regulation by Crc occurs at the post-transcriptional level through a mechanism involving the global RNA-binding protein, Hfq. In this work, the authors employed cryo-EM to investigate the structure of Crc in combination with Hfq and the translation initiation regions of three selected mRNA binding partners, i.e. amiE, estA, and rbsB. The authors solved the structures of all of these complexes and discovered structural polymorphism among the different assemblies, which relied on specific sequence motifs in the mRNAs. Interestingly, the obtained quaternary architectures involved several copies of Crc and Hfq forming higher order repressive complexes. Importantly, the information obtained from the cryo-EM structures is supported by extensive genetic and biochemical data. Taken together, I think this is a high quality manuscript and I have only few comments, which I listed below.

Major point of criticism:
- I have only one major comment, which refers to the stoichiometry of the reported complexes and how this would translate in the availability of Hfq and Crc in the cell. Specifically, Hfq has been reported to be limiting in the cell constantly cycling between different RNP complexes (e.g. Fender et al, Genes Dev., PMID: 21123649) and I wondered how these data can be corroborated with the findings in this manuscript. This question is briefly addressed in the discussion section (lines 428-434), however, it is not clear to me how ribonucleases would initiate turn-over of the complex (would they have access the relevant RNA segments in the first place?).

Our reply: We thank the reviewer for raising this important question, and we note that models must account for the substantial and effective translational repression observed in the presence of succinate. In the 2014 PLoS Genetics paper, repression resulted with only a small increase in Crc. We do not have any data on cycling or details of how ribonuclease would gain access for turn-over, other than

the observation itself that RNAs are indeed degraded upon translation repression by Hfq (Sonnleitner & Bläsi, 2014). We have now dedicated an entire paragraph in the discussion section to the topic of Hfq/Crc availability during/for CCR (lines 449-492). To address these questions further experimentally, we would need an approach to quantify Hfq cycling *in vivo*. These is the topic of a future project.

Minor points of criticism:
- Line 79: suggest to change to "well-studied"
- Fig. 2C: italicize "lacZ"
- Lines 247: I thought this part was a little confusing: if Crc interacts with the phosphate backbone, how is specificity achieved?
- Line 294: italicize "crc"

Our reply: We made the suggested changes to the revised text. Regarding Crc interactions, the specificity is achieved by ARN-motif presentation by Hfq.

Referee #3:

In 2021 the authors had already shown that "Stabilization of Hfq mediated translational repression by the co-repressor Crc in Pseudomonas aeruginosa" (2021, NAR 49, 7075-7087). They demonstrated that in this organism Hfq can collaborate with Crc protein to down-regulate translation of mRNAs that become targets for the degradative machinery. The model proposed in the last figure of this manuscript already proposed that Hfq and Crc could form higher order assemblies.

In this current paper the findings provide structural confirmation of these assemblies in different quaternary structures, and showed that the multi-step assembly process is highly cooperative and probably competes with the ribosomes repressing translation. The structural polymorphism enables selective translational repression of many different target RNAs. It is apparent that completion of higher order assembly enhances translational repression, and that RNA-mediated Hfq-Hfq oligomerization drives this process. The Hfq/Crq assembly is tuned temporally and the resulting translation repression complexes are transient in nature, subject to kinetic competition.

Most of the conclusions are well supported (with exception of the results of the deletion of an hairpin structure). However, the introduction and the discussion only focus on Pseudomonas aeruginosa and ignore many related publications on the roles of Hfq and interaction with other RNAs /proteins. This has to be substantially improved. Below I indicate several publication which should be referred in the introduction and put in context when discussing the roles of Hfq and translation.

Relevant references to be cited in this manuscript:
Trouillon J, Han K, Attrée I, Lory S. The core and accessory Hfq interactomes across Pseudomonas aeruginosa lineages. Nat Commun. 2022 Mar

10;13(1):1258.

Dendooven T, Sinha D, Roeselová A, Cameron TA, De Lay NR, Luisi BF, Bandyra KJ. A cooperative PNPase-Hfq-RNA carrier complex facilitates bacterial riboregulation.
Mol Cell. 2021 Jul 15;81(14):2901-2913.e5.

Bharwad, K. & Rajkumar, S. Rewiring the functional complexity between Crc, Hfq and sRNAs to regulate carbon catabolite repression in Pseudomonas. World J. Microbiol. Biotechnol. 35, 140 (2019).

dos Santos, R. F., Arraiano, C. M. & Andrade, J. M. New molecular interactions broaden the functions of the RNA chaperone Hfq. Curr. Genet. 65, 1313-1319 (2019).

Andrade, J. M., dos Santos, R. F., Chelysheva, I., Ignatova, Z. & Arraiano, C. M. The RNA-binding protein Hfq is important for ribosome biogenesis and affects translation fidelity. EMBO J. 37, e97631 (2018).

Chen J, Gottesman S. Hfq links translation repression to stress-induced mutagenesis in E. coli. Genes Dev. 2017 Jul 1;31(13):1382-1395.

When it is mentioned that Hfq has different interaction surfaces it has to be mentioned the first article which called the attention to this fact:
Zhang A, Schu DJ, Tjaden BC, Storz G, Gottesman S. Mutations in interaction surfaces differentially impact E. coli Hfq association with small RNAs and their mRNA targets. J Mol Biol. 2013 Oct 9;425(19):3678-97.

Our reply: We have revised the introduction and discussion and incorporated the references.

In this manuscript it is not explained the puzzling effect of increasing translation repression when it is deleted a stem loop before the start codon. Please try to give a better explanation taking into account related papers, and refer to them in your discussion:
Ishikawa H, Otaka H, Maki K, Morita T, Aiba H. The functional Hfq-binding module of bacterial sRNAs consists of a double or single hairpin preceded by a U-rich sequence and followed by a 3' poly(U) tail. RNA. 2012 May;18(5):1062-74.

Hopkins, J. F., Panja, S., McNeil, S. A. N. & Woodson, S. A. Effect of salt and RNA structure on annealing and strand displacement by Hfq. Nucleic Acids Res. 37, 6205-6213 (2009).

Our reply: We thank the reviewer for noting these references in regard to the puzzling effect of deleting a stem loop. We believe that this is related to the effects we are seeing in mutating the second putative Hfq binding site in the coding region of the *amiE* transcript, and we have added an explanation to the discussion section and incorporated the suggested references.

These were my major concerns and below I indicate some minor concerns during the reading of the manuscript.

Minor concerns:

1- In the beginning of the introduction (page 3) it refers that only two post-transcriptional systems control gene expression in P. aeruginosa. The sentences have to be rephrased since other important mechanisms are also quite relevant in post-transcriptonal control, such as the action of ribonucleases and the relevance of RNA degradation.

Our reply: We have changed the wording.

2-Line 66 you have to mention that Hfq has also been shown to be in complex with many other proteins (see above some relevant references).

Our reply: We have added a sentence at the end of the paragraph.

3- Line 99- Refer who has discovered the three binding surfaces in Hfq- namely the paper above "Zhang et al 2013".

Our reply: We have added the reference.

4-Line 360- The role of RNA stability/degradation has to be better stressed throughout the text.
Namely it has been considered that respective mRNA levels = relative translational efficiency to account for intrinsic changes in RNA stability.

Our reply: The translational efficiency shown in Figure 5 is the ratio of beta-galactosidase activity to the transcript level. We have not explored RNA degradation in this study, but it has been explored in earlier work by Sonnleitner & Bläsi, 2014. We have changed the text to comment on RNA stability.

5- Try to explain better why to remove an hairpin structure has significantly stronger translation repression.

Our reply: We have added text to the discussion section regarding this point described above.

6- Fig5- It is said that mutations are indicated in green, but that is not clear.

Our reply: We have changed the text of the figure legend to improve the clarity. The changes to the sequence, i.e. hairpin removal and mutagenesis of the Hfq3 binding site, are depicted in green.

1st Revision - Editorial Decision **19th Oct 2022**

Thank you again for submitting your revised manuscript and providing the dataset access. We have now received comments from two of the initial referees (please see below) and I am happy to say they now support publication. Therefore, I would ask you to please resolve a number of editorial issues that are listed in detail below. Please use the document that the data editors have added their comments to for any changes (see below), or clearly mark all changes you are making to the last version of your manuscript. If you have any further questions regarding the specific points listed below or the final manuscript version, please feel free to contact me. Once these issues are resolved, we will be happy to formally accept the manuscript for publication.

Referee #1:

This data-rich manuscript presents a set of structures highlighting the multiple ways in which Hfq and Crc collaborate to carry out translational repression in Pseudomonas. The revised manuscript addresses and clarifies previous questions, and more clearly discusses mutations that help to support the in vivo relevance of the structures.

Referee #2:

The authors have adequately addressed my previous concerns in the revised version of their manuscript and I have no further comments.

Thank you again for submitting the final revised version of your manuscript and addressing the remaining points. I am pleased to inform you that we have now accepted it for publication in The EMBO Journal.

EMBO Press Author Checklist

Corresponding Author Name: Ben Luisi	
Journal Submitted to: EMBO J	
Manuscript Number: EMBOJ-2022-111129R	

USEFUL LINKS FOR COMPLETING THIS FORM
The EMBO Journal - Author Guidelines
EMBO Reports - Author Guidelines
Molecular Systems Biology - Author Guidelines
EMBO Molecular Medicine - Author Guidelines

Reporting Checklist for Life Science Articles (updated January 2022)
This checklist is adapted from Materials Design Analysis Reporting (MDAR) Checklist for Authors. MDAR establishes a minimum set of requirements in transparent reporting in the life sciences (see Statement of Task: 10.31222/osf.io/9sm4x). Please follow the journal's guidelines in preparing your manuscript.
Please note that a copy of this checklist will be published alongside your article.

Abridged guidelines for figures
1. Data
The data shown in figures should satisfy the following conditions:
- the data were obtained and processed according to the field's best practice and are presented to reflect the results of the experiments in an accurate and unbiased manner.
- ideally, figure panels should include only measurements that are directly comparable to each other and obtained with the same assay.
- plots include clearly labeled error bars for independent experiments and sample sizes. Unless justified, error bars should not be shown for technical replicates.
- if n<5, the individual data points from each experiment should be plotted. Any statistical test employed should be justified.
- Source Data should be included to report the data underlying figures according to the guidelines set out in the authorship guidelines on Data Presentation.

2. Captions
Each figure caption should contain the following information, for each panel where they are relevant:
- a specification of the experimental system investigated (eg cell line, species name).
- the assay(s) and method(s) used to carry out the reported observations and measurements.
- an explicit mention of the biological and chemical entity(ies) that are being measured.
- an explicit mention of the biological and chemical entity(ies) that are altered/varied/perturbed in a controlled manner.
- the exact sample size (n) for each experimental group/condition, given as a number, not a range;
- a description of the sample collection allowing the reader to understand whether the samples represent technical or biological replicates (including how many animals, litters, cultures, etc.).
- a statement of how many times the experiment shown was independently replicated in the laboratory.
- definitions of statistical methods and measures:
  - common tests, such as t-test (please specify whether paired vs. unpaired), simple χ2 tests, Wilcoxon and Mann-Whitney tests, can be unambiguously identified by name only, but more complex techniques should be described in the methods section;
  - are tests one-sided or two-sided?
  - are there adjustments for multiple comparisons?
  - exact statistical test results, e.g., P values = x but not P values < x;
  - definition of 'center values' as median or average;
  - definition of error bars as s.d. or s.e.m.

Please complete ALL of the questions below.
Select "Not Applicable" only when the requested information is not relevant for your study.

Materials

**Newly Created Materials**	**Information included in the manuscript?**	**In which section is the information available?** (Reagents and Tools Table, Materials and Methods, Figures, Data Availability Section)
New materials and reagents need to be available; do any restrictions apply?	Not Applicable	

**Antibodies**	**Information included in the manuscript?**	**In which section is the information available?** (Reagents and Tools Table, Materials and Methods, Figures, Data Availability Section)
For **antibodies** provide the following information:<br>- Commercial antibodies: RRID (if possible) or supplier name, catalogue number and or/clone number<br>- Non-commercial: RRID or citation	Not Applicable	

**DNA and RNA sequences**	**Information included in the manuscript?**	**In which section is the information available?** (Reagents and Tools Table, Materials and Methods, Figures, Data Availability Section)
**Short novel DNA or RNA including primers, probes**: provide the sequences.	Yes	Materials and Methods

**Cell materials**	**Information included in the manuscript?**	**In which section is the information available?** (Reagents and Tools Table, Materials and Methods, Figures, Data Availability Section)
**Cell lines:** Provide species information, strain. Provide accession number in repository **OR** supplier name, catalog number, clone number, and/**OR** RRID.	Not Applicable	
**Primary cultures:** Provide species, strain, sex of origin, genetic modification status.	Not Applicable	
Report if the cell lines were recently **authenticated** (e.g., by STR profiling) and tested for mycoplasma contamination.	Not Applicable	

**Experimental animals**	**Information included in the manuscript?**	**In which section is the information available?** (Reagents and Tools Table, Materials and Methods, Figures, Data Availability Section)
**Laboratory animals or Model organisms:** Provide species, strain, sex, age, genetic modification status. Provide accession number in repository **OR** supplier name, catalog number, clone number, **OR** RRID.	Not Applicable	
**Animal observed in or captured from the field:** Provide species, sex, and age where possible.	Not Applicable	
Please detail **housing and husbandry conditions**.	Not Applicable	

**Plants and microbes**	**Information included in the manuscript?**	**In which section is the information available?** (Reagents and Tools Table, Materials and Methods, Figures, Data Availability Section)
**Plants:** provide species and strain, ecotype and cultivar where relevant, unique accession number if available, and source (including location for collected wild specimens).	Not Applicable	
**Microbes:** provide species and strain, unique accession number if available, and source.	Yes	Materials and Methods

**Human research participants**	**Information included in the manuscript?**	**In which section is the information available?** (Reagents and Tools Table, Materials and Methods, Figures, Data Availability Section)
If collected and within the bounds of privacy constraints report on age, sex and gender or ethnicity for all study participants.	Not Applicable	

**Core facilities**	**Information included in the manuscript?**	**In which section is the information available?** (Reagents and Tools Table, Materials and Methods, Figures, Data Availability Section)
If your work benefited from core facilities, was their service mentioned in the acknowledgments section?	Yes	ACKNOWLEDGEMENT

Design

Study protocol	Information included in the manuscript?	In which section is the information available? (Reagents and Tools Table, Materials and Methods, Figures, Data Availability Section)
If study protocol has been **pre-registered, provide DOI in the manuscript**. For clinical trials, provide the trial registration number **OR** cite DOI.	Not Applicable	
Report the **clinical trial registration number** (at ClinicalTrials.gov or equivalent), where applicable.	Not Applicable	

Laboratory protocol	Information included in the manuscript?	In which section is the information available? (Reagents and Tools Table, Materials and Methods, Figures, Data Availability Section)
Provide DOI OR other citation details if **external detailed step-by-step protocols** are available.	Not Applicable	

Experimental study design and statistics	Information included in the manuscript?	In which section is the information available? (Reagents and Tools Table, Materials and Methods, Figures, Data Availability Section)
Include a statement about **sample size** estimate even if no statistical methods were used.	Yes	Figure legend
Were any steps taken to minimize the effects of subjective bias when allocating animals/samples to treatment (e.g. **randomization procedure**)? If yes, have they been described?	Not Applicable	
Include a statement about **blinding** even if no blinding was done.	Not Applicable	
Describe **inclusion/exclusion criteria** if samples or animals were excluded from the analysis. Were the criteria pre-established?<br><br>If sample or data points were omitted from analysis, report if this was due to attrition or intentional exclusion and provide justification.	Not Applicable	
For every figure, are **statistical tests** justified as appropriate? Do the data meet the assumptions of the tests (e.g., normal distribution)? Describe any methods used to assess it. Is there an estimate of variation within each group of data? Is the variance similar between the groups that are being statistically compared?	Yes	Figure legend

Sample definition and in-laboratory replication	Information included in the manuscript?	In which section is the information available? (Reagents and Tools Table, Materials and Methods, Figures, Data Availability Section)
In the figure legends: state number of times the experiment was **replicated** in laboratory.	Yes	Figures
In the figure legends: define whether data describe **technical or biological replicates**.	Yes	Figures

Ethics

Ethics	Information included in the manuscript?	In which section is the information available? (Reagents and Tools Table, Materials and Methods, Figures, Data Availability Section)
Studies involving **human participants**: State details of **authority granting ethics approval** (IRB or equivalent committee(s), provide reference number for approval.	Not Applicable	
Studies involving **human participants**: Include a statement confirming that **informed consent** was obtained from all subjects and that the experiments conformed to the principles set out in the WMA Declaration of Helsinki and the Department of Health and Human Services Belmont Report.	Not Applicable	
Studies involving **human participants:** For publication of **patient photos**, include a statement confirming that consent to publish was obtained.	Not Applicable	
Studies involving experimental **animals**: State details of **authority granting ethics approva**l (IRB or equivalent committee(s), provide reference number for approval. Include a statement of compliance with ethical regulations.	Not Applicable	
Studies involving **specimen and field samples:** State if relevant **permits** obtained, provide details of authority approving study; if none were required, explain why.	Not Applicable	

Dual Use Research of Concern (DURC)	Information included in the manuscript?	In which section is the information available? (Reagents and Tools Table, Materials and Methods, Figures, Data Availability Section)
Could your study fall under dual use research restrictions? Please check biosecurity documents and list of **select agents and toxins** (CDC): https://www.selectagents.gov/sat/list.htm	Not Applicable	
If you used a select agent, is the security level of the lab appropriate and reported in the manuscript?	Not Applicable	
If a study is subject to dual use research of concern regulations, is the name of the **authority granting approval and reference number** for the regulatory approval provided in the manuscript?	Not Applicable	

Reporting

The MDAR framework recommends adoption of discipline-specific guidelines, established and endorsed through community initiatives. Journals have their own policy about requiring specific guidelines and recommendations to complement MDAR.

Adherence to community standards	Information included in the manuscript?	In which section is the information available? (Reagents and Tools Table, Materials and Methods, Figures, Data Availability Section)
State if relevant guidelines or checklists (e.g., **ICMJE, MIBBI, ARRIVE, PRISMA**) have been followed or provided.	Not Applicable	
For **tumor marker prognostic studies,** we recommend that you follow the **REMARK** reporting guidelines (see link list at top right). See author guidelines, under 'Reporting Guidelines'. Please confirm you have followed these guidelines.	Not Applicable	
For **phase II and III randomized controlled trials**, please refer to the **CONSORT** flow diagram (see link list at top right) and submit the CONSORT checklist (see link list at top right) with your submission. See author guidelines, under 'Reporting Guidelines'. Please confirm you have submitted this list.	Not Applicable	

Data Availability

Data availability	Information included in the manuscript?	In which section is the information available? (Reagents and Tools Table, Materials and Methods, Figures, Data Availability Section)
Have **primary datasets** been deposited according to the journal's guidelines (see 'Data Deposition' section) and the respective accession numbers provided in the Data Availability Section?	Yes	Data Availability Section: PDB and EMDB
Were **human clinical and genomic datasets** deposited in a public access-controlled repository in accordance to ethical obligations to the patients and to the applicable consent agreement?	Not Applicable	
Are **computational models** that are central and integral to a study available without restrictions in a machine-readable form? Were the relevant accession numbers or links provided?	Not Applicable	
If publicly available data were reused, provide the respective **data citations in the reference list**.	Not Applicable	
